# Comparative efficacy and safety of GLP-1 receptor agonists for weight reduction: A model-based meta-analysis of placebo-controlled trials

**DOI:** 10.1016/j.obpill.2025.100162

**Published:** 2025-01-30

**Authors:** Haoyang Guo, Juan Yang, Jihan Huang, Ling Xu, Yinghua Lv, Yexuan Wang, Jiyuan Ren, Yulin Feng, Qingshan Zheng, Lujin Li

**Affiliations:** a Center for Drug Clinical Research, Shanghai University of Traditional Chinese Medicine, No.1200 Cailun Road, Shanghai, 201203, China; b State Key Laboratory of Integration and Innovation of Classic Formula and Modern Chinese Medicine (Shanghai University of Traditional Chinese Medicine), China

**Keywords:** GLP-1RA, Model-based meta-analysis, Obesity, Weight reduction

## Abstract

**Aim:**

Obesity is a global epidemic. The FDA has approved glucagon-like peptide-1 (GLP-1) receptor agonists such as Liraglutide, Semaglutide, and the GLP-1/gastric inhibitory polypeptide (GIP) dual agonist Tirzepatide for the treatment of obesity. Clinical trials of GLP-1/GIP/glucagon(GCG) triple agonists are ongoing. This study compared the efficacy and safety profiles of different GLP-1 receptor agonists (GLP-1RAs) for weight reduction and explored the related influencing factors, providing quantitative information for the development of GLP-1RAs and their clinical use.

**Methods:**

This systematic review of public databases included placebo-controlled randomized clinical trials of GLP-1RAs. Time-course, dose-response, and covariate models were used to describe the efficacy characteristics and influencing factors of different GLP-1RAs. Subgroup analyses were performed to explore efficacy differences in receptor specificity. Meta-analyses compared the incidence of adverse event and dropout rates among different GLP-1RAs.

**Results:**

Fifty-five studies involving 16,269 participants and 12 GLP-1RAs were included. Six drugs showed significant dose-response relationships. The maximum weight reduction effect ranged from 4.25 kg (Liraglutide) to 22.6 kg (Retatrutide). Reported onset times ranged from 6.4 weeks (Orforglipron) to 19.5 weeks (Tirzepatide). At 52 weeks, weight reduction effects were 7.03 kg, 11.07 kg, and 24.15 kg for mono-agonists, dual-agonists, and tri-agonists, respectively. There was a significant negative correlation in the exponential pattern between age and weight reduction effect, whereas baseline weight and BMI had no significant impact. Common adverse events of GLP-1RAs, reported in the literature include nausea, vomiting, diarrhea, and constipation, with a significantly higher incidence of nausea than that of placebo.

**Conclusions:**

This study provides a quantitative evaluation of the efficacy and safety of GLP-1RAs and offers valuable insights into the assessment of new drugs for weight reduction.

## Highlights

• Offers a detailed comparison of the efficacy, potential influencing factors and safety profiles among various GLP-1RAs.

## Graphical abstract

**Figure:**
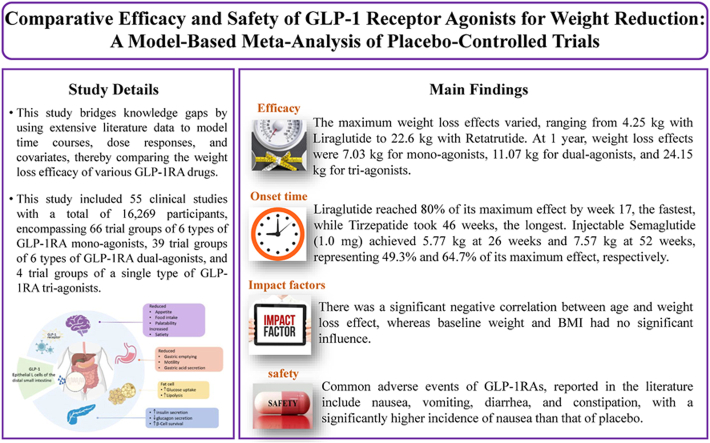

## 1 Introduction

Obesity is a major global epidemic, defined by the World Health Organization (WHO) as “an excessive accumulation of body fat that may impair health” [1,2]. It is linked to adverse metabolic changes and chronic diseases such as type 2 diabetes, coronary artery disease, neurological disorders, and certain cancers [3,4]. Recent statistics indicate that overweight/obesity continues to increase globally, with the overweight population reaching 2 billion, or 30 % of the world's population [5]. According to the WHO, approximately 1.9 billion adults are overweight, with over 650 million with obesity [6]. Reducing weight by 5 % or more significantly improves obesity-related health complications [7]. Key strategies for managing obesity include improving diet and increasing physical activity [8]. For those unable to control their weight through lifestyle changes or additional comorbidities, pharmacological treatment is often essential [9,10].

In obesity pharmacological research, glucagon-like peptide-1 (GLP-1) promotes insulin secretion, inhibits glucagon release, slows gastric emptying, reduces appetite, and decreases food intake [11,12]. Glucose-dependent insulinotropic polypeptide (GIP) enhances insulin sensitivity and regulates lipid metabolism, while glucagon (GCG) increases satiety, energy expenditure, hepatic glucose production, insulin secretion, and lipid breakdown [13,14]. Dual agonists for GLP-1/GIP or GLP-1/GCG receptors, and triple agonists for GLP-1/GIP/GCG receptors have been developed to enhance weight reduction and metabolic outcomes [15]. The FDA has approved GLP-1 receptor agonists such as Liraglutide, Semaglutide, and the GLP-1/GIP dual agonist Tirzepatide for the treatment of obesity [[16], [17], [18]]. Clinical trials for GLP-1/GIP/GCG triple agonists are ongoing [19]. However, quantitative comparisons of the efficacy of these GLP-1 receptor agonist drugs in obesity treatment, particularly between mono-, dual-, and triple-agonists, are still lacking.

This study addresses these knowledge gaps by collecting and analyzing literature to establish time-course, dose-response, and covariate models to compare the therapeutic characteristics of different GLP-1RA drugs [20]. The results provide quantitative data for the evaluation of new drugs and, optimization of treatment strategies for people with obesity.

## 2 Methods

### 2.1 Research criteria and eligibility

A literature search was conducted in the PubMed and Embase databases, covering the period from their inception to January 20, 2024. Search terms included “obesity” and “GLP-1,” and the search was limited to clinical trials published in English. Details of the search strategy are provided in the Supplementary Table S1.

Inclusion criteria for the literature were [1]: trials designed as randomized, double-blind, placebo-controlled studies [2]; interventions involving GLP-1RA treatment [3]; participants aged ≥18 years who were overweight or have obesity, defined as having a BMI ≥25 kg/m^2^ (or ≥23 kg/m^2^ for Japanese and ≥24 kg/m2 for Chinese) [4]; studies reporting changes in patient weight from baseline.

### 2.2 Data extraction and quality assessment

Microsoft Excel was used as the database input template to extract the following information from each eligible study: literature characteristics (DOI, authors, publication year, and clinical trial registration number); study design (sample size, dosing regimen, dosage, and duration of treatment); participant characteristics (age, percentage of males, baseline weight, and baseline body mass index); and clinical outcomes (changes in weight from baseline at each follow-up time point). In cases where studies reported both intention-to-treat (ITT) and per-protocol (PP) results, the ITT analysis results were prioritized for inclusion for conservatism. If the data in the publications were presented in graphical form, Engauge Digitizer (version 11.3) was used for data extraction. The error margin for data extraction from the graphs was kept below 2 %; if it exceeded this threshold, the data were re-extracted.

The quality of the included studies was assessed using the Cochrane Risk of Bias Tool 2.0 [21]. The specific information is detailed in Supplementary Table S5 and Fig. S2.

### 2.3 Model establishment

To account for heterogeneity in dietary and exercise controls across different trials, this study modeled the pure effect of drug-induced weight reduction, represented as the change in weight relative to baseline (ΔΔWeight) in the drug group compared to the placebo group.

This study established time-course, dose-response, and covariate models for each drug. The potential covariates examined included age, male ratio, baseline weight, and baseline BMI. Data were fitted using a nonlinear mixed-effects model. The detailed methods for model construction and evaluation are provided in Supplementary Methods 1.

### 2.4 Typical efficacy comparison

Based on the final model, simulations of the typical time-course of weight reduction by different drugs at various levels of covariates were conducted. Additionally, we conducted subgroup analyses based on receptor specificity to explore the differences in weight reduction effects among mono-agonists, dual-agonists, and tri-agonists.

The subgroup analysis included the following steps. First, individual estimates of model parameters for each drug group and their standard errors were obtained using data output by the NONMEM software. If the influence of covariates was involved, adjustments were made through the inverse operation of the covariate formula to mitigate the effects of uneven distribution across different studies owing to covariate effects. Next, data synthesis was performed using a meta-analysis for single-arm trials, categorized by subgroup, to determine the overall mean and 95 % CIs for each subgroup. Finally, parameters from the distribution of pharmacodynamic parameters in each subgroup were randomly selected, and drug effects at different time points were calculated. This process was repeated 10,000 times to estimate the median and 95 % confidence interval (CIs) of the drug effects at each time point for each subgroup.

### 2.5 Dropout and adverse event analysis

As most of the included studies only reported dropout rates and the incidence of adverse events at the endpoint, we were unable to establish a time-course model for them. This study aimed to summarize these indicators through a meta-analysis. The relative risk (RR) of the drug relative to the placebo along with its 95 % CIs were calculated. Heterogeneity among studies was assessed using the I^2^ statistic, with an I^2^ value of 50 % or higher indicating obvious heterogeneity. When heterogeneity was obvious (I^2^ ≥ 50 %), a random-effects model was used to summarize the RRs; otherwise, a fixed-effect model was applied.

### 2.6 Analysis software

Modeling and simulation were conducted using NONMEM 7.4 (ICON Development Solutions, USA), and model parameter estimation was performed using the First-Order Conditional Estimation method (FOCE-I). Data analysis and graphical visualization were performed using R 4.3.1 (The R Foundation for Statistical Computing, Vienna, Austria).

## 3 Results

### 3.1 Characteristics of the included studies

**Fig. 1:**
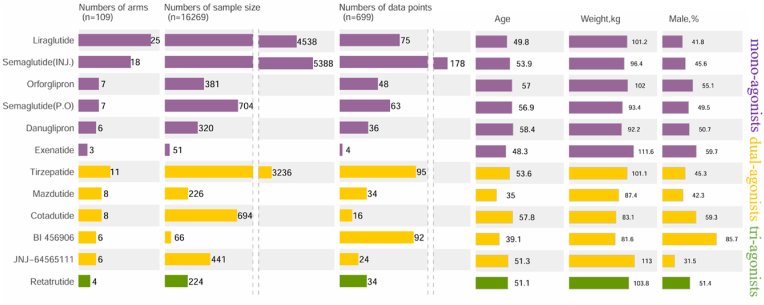
Characteristics of the included studies.

A total of 1789 articles were retrieved from PubMed and Embase databases. Following strict inclusion and exclusion criteria, 55 articles were included in the analysis. Among these publications, studies on mono-agonists included 18 on Liraglutide, 11 on injectable Semaglutide, two on oral Semaglutide, three on Exenatide, two on Danuglipron, and two on Orforglipron. Research on dual-agonists comprised: four articles on Cotadutide, four on Tirzepatide, three on Mazdutide, two on JNJ-64565111, two on BI 456906, and two on Danuglipron. Studies on tri-agonists included two articles on Retatrutide. A flowchart of the literature selection process is provided in Supplementary Fig. S1, and specific data characteristics are provided in Fig. 1 and Supplementary Table S2.

The selected 55 studies included a total of 16,269 participants, with an average age range from 29.5 to 64.7 years, and a median age of 53.6 years. The proportion of male participants ranged from 19.1 % to 100.0 %, with a median of 51.6 %. Average baseline weight ranged from 72.2 to 121 kg, with a median weight of 95.8 kg; average baseline Body Mass Index (BMI) ranged from 24.1 to 45.1 kg/m^2^, with a median of 33.9 kg/m^2^. Treatment duration varied from 6 weeks to 104 weeks, with a median duration of 26 weeks. The detailed demographic and clinical characteristics of the participants are provided in Supplementary Tables S3 and S4.

Among the 55 studies included, 22 (40 %) were assessed as having a moderate risk of bias, whereas the remaining 33 (60 %) were considered to have a low risk of bias (Supplementary Table S5 and Fig. S2).

### 3.2 Model establishment and evaluation

The estimated parameters for the final model are presented in Supplementary Table S6. This study identified significant dose-response relationships for Cotadutide, Danuglipron, JNJ-64565111, Retatrutide, Orforglipron, and injectable Semaglutide (Equations Equation 1, Equation 2, Equation 3, Equation 4, Equation 5, Equation 6), with effective doses (doses achieving 50 % of the Emax value) of 0.219 mg, 80 mg, 6.73 mg, 4 mg, 14.6 mg, and 0.384 mg, respectively. Additionally, during covariate selection, age significantly affected the Emax value (Equation (7)) (Supplementary Fig. S5); an increase in the average age from 40 to 50 years was associated with a 26.2 % decrease in the Emax value.

Equation 1 $$E_{\max\;\_Cotadutide}=\frac{{-10.5\times Dose}_{Cotadutide}}{0.219+{Dose}_{Cotadutide}}$$

Equation 2 $$E_{\max\;\_Danuglipron}=\frac{{-9.29\times Dose}_{Danuglipron}}{80+{Dose}_{Danuglipron}}$$

Equation 3 $$E_{\max\;\_JNJ-6456111}=\frac{{-18.6\times Dose}_{JNJ-6456111}}{6.73+{Dose}_{JNJ-6456111}}$$

Equation 4 $$E_{\max\;\_Retatrutide}=\frac{{-22.6\times Dose}_{Retatrutide}}{4+{Dose}_{Retatrutide}}$$

Equation 5 $$E_{\max\;\_Orforglipron}=\frac{{-14.7\times Dose}_{Orforglipron}}{14.6+{Dose}_{Orforglipron}}$$

Equation 6 $$E_{\max\;\_Semaglutide\_injection}=\frac{{-11.7\times Dose}_{Semaglutide\_injection}}{0.384+{Dose}_{Semaglutide\_injection}}$$

Equation 7 $$E_{\max}=E_{\max,typical}\times e^{-0.0304\times \left(Age-53.6\right)}$$

The Emax values for 12 GLP-1RA drugs ranged from 4.25 kg to 22.6 kg, with Retatrutide exhibiting the highest Emax value and Liraglutide the lowest. Owing to the limited number of time points available for some drugs, it was not possible to estimate the k values individually for each drug. Therefore, this study employed Bayesian feedback combined with single-arm meta-analysis to estimate the onset time (the time required to achieve 50 % of the E_max_ value, ET50 = 0.693/k) for each drug. Orforglipron had the fastest onset at 6.4 weeks, whereas Tirzepatide had the slowest at 19.5 weeks (Supplementary Fig. S3).

**Fig. 2:**
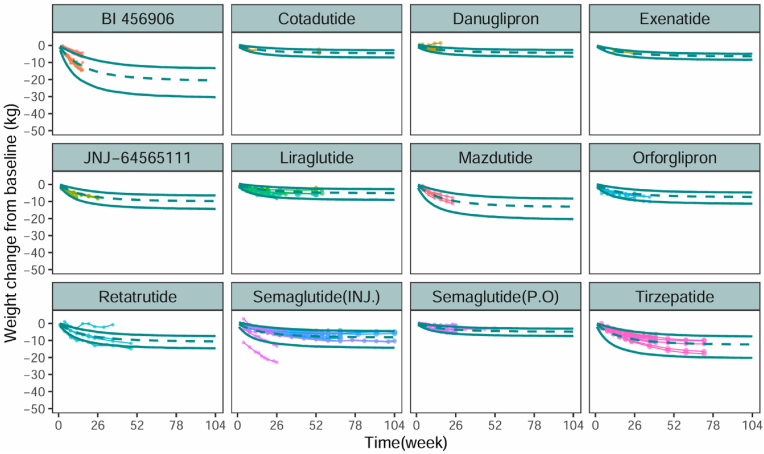
**Prediction-corrected visual predictive check of the final mode** Observed efficacy data are represented by points, with symbol size reflecting sample size. Points connected by a line originate from the same study arm, while points sharing the same color belong to the same study. The green lines indicate the model-predicted 5th, 50th, and 95th percentiles of efficacy. Most of the observed efficacy data fall within the 90 % confidence interval predicted by the model, indicating that the model has good predictive ability. (For interpretation of the references to color in this figure legend, the reader is referred to the Web version of this article.)

The goodness-of-fit plots for the model indicated that the model fit the observed data well, with no evident bias (Supplementary Fig. S4). The median values of the parameters estimated using the SIR method were very close to those estimated from the final model (Supplementary Table S6), suggesting that the parameter estimates were robust. The VPC plots (Fig. 2) showed that most observed efficacy values fell within the 95 % CI of the model predictions, indicating that the model had a good predictive capability.

### 3.3 Typical efficacy comparison

**Fig. 3:**
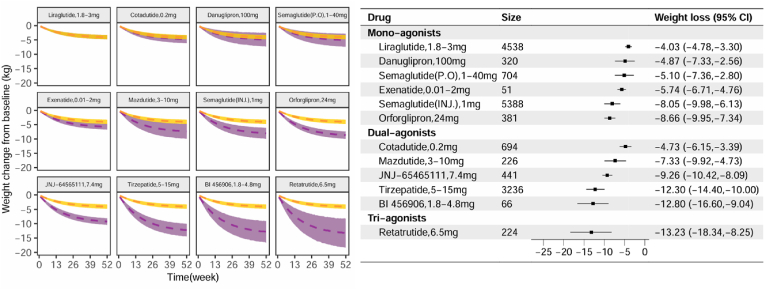
**Distribution of typical pure effect values for weight reduction at week 52 for each drug** The graph on the left represents the 95 % CIs of the typical pure effect of weight reduction over 52 weeks, with the yellow stripe indicating Liraglutide and the purple stripe representing other drugs. The lack of overlap between the two stripes indicates a significant difference in efficacy between the two drugs. Points on the right indicate the typical values of the pure effect of 52-week weight reduction for each drug, with error bars denoting 95 % CIs of the estimates. (For interpretation of the references to color in this figure legend, the reader is referred to the Web version of this article.)

Based on the final model parameters, we simulated the time-course distribution of weight reduction for each GLP-1RA drug. The results indicate that the weight reduction effects of these GLP-1RA drugs at 52 weeks can be broadly categorized into three tiers: Liraglutide (1.8–3 mg), oral Semaglutide (1–40 mg), Exenatide (0.01–2 mg), Mazdutide (3–10 mg), Cotadutide (0.2 mg) and Danuglipron (100 mg) exhibited weight reduction effects ranging from 4 to 7 kg; injectable Semaglutide (1.0 mg), Orforglipron (24 mg) and JNJ-64565111 (7.4 mg) showed effects ranging from 8 to 9 kg; while Tirzepatide (5–15 mg), BI 456906 (1.8–4.8 mg) and Retatrutide (6.5 mg) demonstrated weight reduction effects exceeding 13 kg (Fig. 3).

**Fig. 4:**
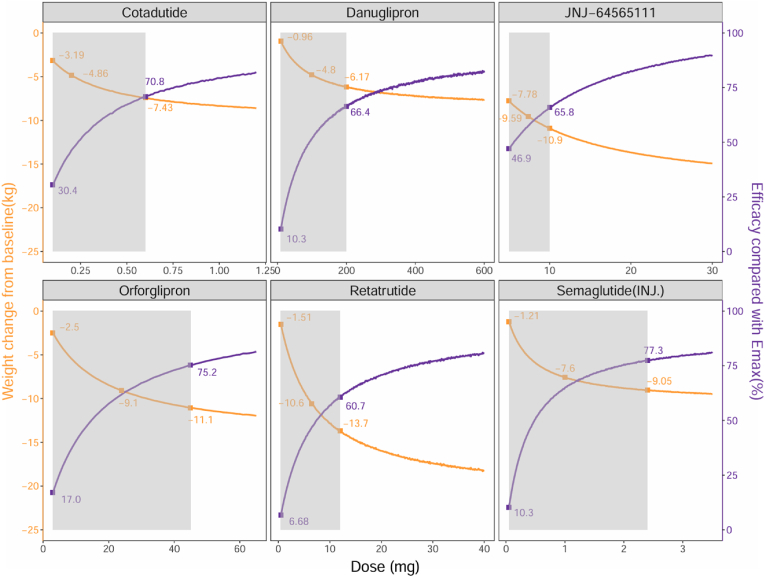
**Dose-response characteristics of each drug at 52 weeks** The orange line represents the typical dose-response curve of the drug, while the purple line shows the percentage of efficacy at each dose relative to the maximum effect of each drug. Values exceeding 80 % suggest that the dosage of the drug has approached or reached its efficacy plateau. The shaded gray area represents the range of doses administered in the included studies, while the plotted points indicate the maximum, median, and minimum drug doses observed across the studies. (For interpretation of the references to color in this figure legend, the reader is referred to the Web version of this article.)

This study found a clear dose-response relationship for Cotadutide, Danuglipron, JNJ-64565111, Retatrutide, Orforglipron, and injectable Semaglutide. We simulated the dose-response weight reduction effects at their lowest, median, and highest doses. For example, with injectable Semaglutide, at doses of 0.05 mg, 1.0 mg, and 2.4 mg, the weight reduction effects at 52 weeks were1.21 kg, 7.6 kg, and 9.05 kg, respectively (Fig. 4).

**Fig. 5:**
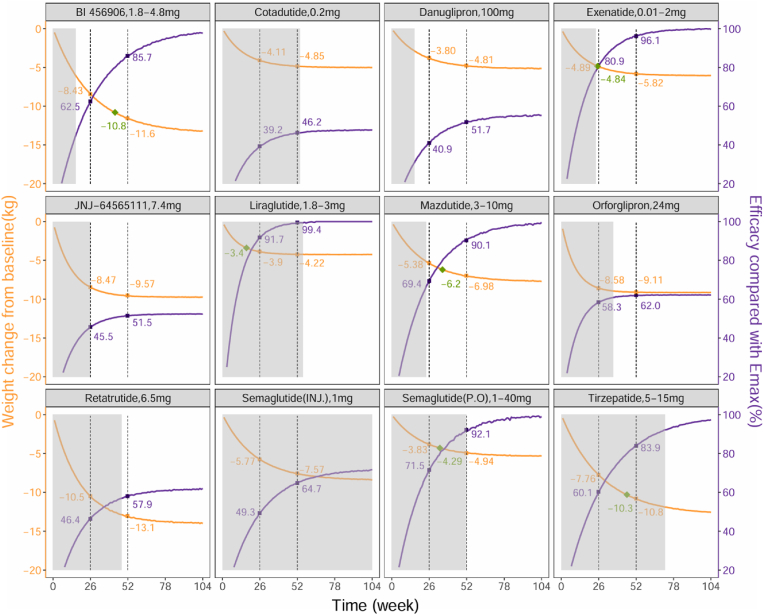
**Time-effect characteristics of each drug** The orange line represents the typical time-course curve of the drug, while the purple line represents the percentage of the efficacy at each time point relative to the maximum effect of each drug. Values exceeding 80 % suggest that the treatment duration has approached or reached its efficacy plateau. The shaded gray area represents the range of treatment duration in the included studies, while the orange points indicate the effect values at 26 weeks and 52 weeks. The green diamond points represent the efficacy values that reach the efficacy plateau (80 % of maximum effect). (For interpretation of the references to color in this figure legend, the reader is referred to the Web version of this article.)

This study simulated the time-course processes for 12 drugs. The results indicated that Liraglutide exhibited the fastest onset, reaching an efficacy plateau (80 % of the E_max_ value) by week 17, whereas Tirzepatide showed the slowest onset, requiring 46 weeks to reach the efficacy plateau. Taking injectable Semaglutide (1.0 mg) as an example, the efficacy at 26 weeks, and 52 weeks was 5.77 kg, and 7.57 kg, respectively, representing 49.3 %, and 64.7 % of its maximum effect (Fig. 5).

### 3.4 The impact of age on weight reduction efficacy

**Fig. 6:**
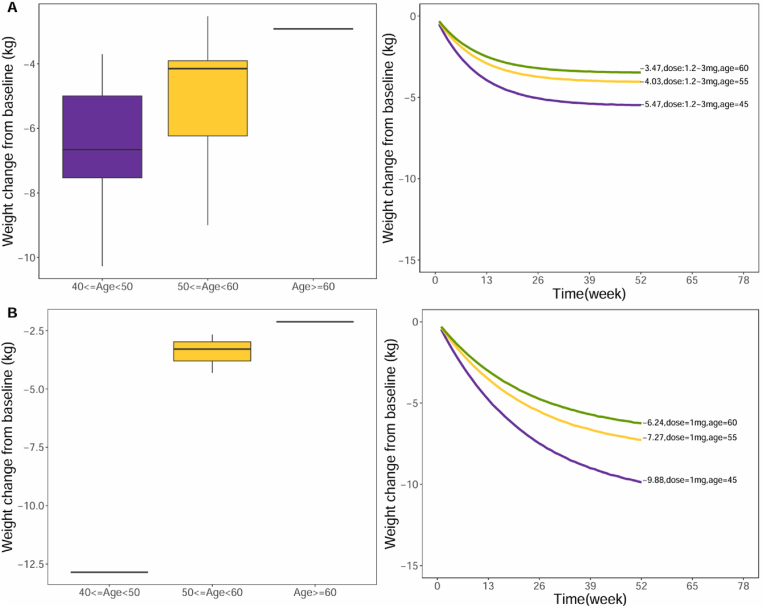
**Impact of age on the weight reduction effects of Liraglutide (A) and Semaglutide (B)** The box plot on the left displays the observed weight reduction values for Liraglutide (A) and Semaglutide (B) across different age groups. The figure on the right illustrates the model-predicted typical values of weight reduction for Liraglutide (A) and Semaglutide (B) at various age levels.

Covariate analysis identified age as a significant factor affecting the weight reduction efficacy of GLP-1RA drugs. This study simulated the weight reduction effects of the drugs across three age groups (45, 55, and 60 years). Taking injectable Semaglutide (1.0 mg) as an example, the weight reduction effects at 52 weeks for subjects aged 45, 55, and 60 were 9.88 kg, 7.27 kg, and 6.24 kg, respectively, with the latter being 3.64 kg lower than the former (Fig. 6).

### 3.5 Subgroup analysis of receptor specificity

The 12 GLP-1RA drugs were categorized based on receptor specificity into mono-agonists, dual-agonists, and triple-agonists. The results indicated that at 52 weeks, the weight reduction effects of mono-agonists, dual-agonists, and triple-agonists were 7.03 kg, 11.07 kg, and 24.15 kg, respectively.

### 3.6 Dropout and adverse events analysis

An analysis was conducted on the total dropout rates for the 12 types of GLP-1RA drugs. The results (Table 1) indicate that the dropout risks for injectable Semaglutide, Liraglutide, Mazdutide, Retatrutide and Tirzepatide were significantly lower than those for the placebo, whereas Orforglipron, oral Semaglutide, Danuglipron, BI 456906, Cotadutide and JNJ-64565111 had significantly higher dropout risks than the placebo.

**Table 1:** Dropout rates and incidence of adverse reactions for each drug.

Drug	RR-Dropout	RR-Constipation	RR-Nausea	RR-Diarrhea	RR-Vomiting
**Mono-agonists**					
**Semaglutide(INJ.)**	0.82(0.69, 0.97)	2.32(1.7, 3.12)	2.66(2.38, 2.97)	1.77(1.59, 1.98)	3.22(2.7, 3.84)
**Semaglutide(P.O)**	1.87(1.14, 3.09)	1.86(0.86, 4.02)	5.34(2.5, 11.4)	1.31(0.54, 3.19)	2.64(1.14, 6.14)
**Danuglipron**	1.82(0.99, 3.36)	0.87(0.04, 18.8)	1.44(0.59, 3.52)	1.08(0.42, 2.77)	1.31(0.46, 3.69)
**Exenatide**	1(0.3, 3.39)	1(0.02, 47.3)	7(0.39, 124.5)	3(0.13, 68.1)	3(0.13, 68.1)
**Liraglutide**	0.84(0.78, 0.92)	2.29(1.95, 2.68)	2.72(2.44, 3.05)	2.05(1.77, 2.37)	0.25(0.03, 0.47)
**Orforglipron**	1.41(0.96, 2.05)	10.4(2.47, 44.1)	10.1(0.87, 117.7)	5.56(0.74, 41.6)	8.81(2.04, 38)
**Overall**	0.94(0.87, 1)	2.29(2.05, 2.56)	2.75(2.55, 2.98)	1.88(1.72, 2.05)	3.26(2.85, 3.73)
**Dual-agonists**					
**Tirzepatide**	0.66(0.49, 0.87)	2.71(2.02, 3.66)	2.71(2.17, 3.39)	2.17(1.73, 2.72)	3.62(2.4, 5.46)
**BI 456906**	2.87(0.81, 10.2)	3.6(0.47, 27.4)	2.83(0.71, 11.4)	1.2(0.43, 3.39)	5.44(0.78, 38.1)
**Mazdutide**	0.95(0.54, 1.65)	1.47(0.18, 11.9)	2.51(1.04, 6.08)	1.22(0.67, 2.21)	4.04(1.3, 12.6)
**Cotadutide**	1.68(1.01, 2.81)	2.16(0.73, 6.43)	5.13(1.72, 15.3)	1.3(0.4, 4.21)	4.86(1.4, 16.9)
**JNJ-65465111**	3.23(2.29, 4.56)	2.05(0.8, 5.27)	4.18(1.58, 11.1)	2.05(0.8, 5.27)	21.6(2.95, 157.9)
**Overall**	1.26(0.92, 1.72)	2.61(2, 3.42)	2.94(2.41, 3.58)	2(1.64, 2.44)	4.3(3.02, 6.12)
**Tri-agonists**					
**Retatrutide**	0.57(0.4, 0.83)	2.23(0.7, 7.05)	1.18(0.56, 2.52)	0.73(0.31, 1.74)	1.32(0.3, 5.75)

Common adverse events associated with GLP-1RA drugs include nausea, vomiting, diarrhea, and constipation. The incidence of nausea for most GLP-1RA drugs was significantly higher than that for placebo, particularly for Orforglipron and Exenatide, with relative risks (RR) of 10.1 and 7, respectively. Regarding vomiting, except for Liraglutide, which was lower than the placebo, the incidence rates for other GLP-1RA drugs were similar to or higher than those for the placebo. The risks of diarrhea was significantly higher for JNJ-64565111, Exenatide, Liraglutide, Orforglipron and Tirzepatide than for placebo, with Orforglipron having an RR of 5.56. In terms of constipation, except for Danuglipron, which was lower than the placebo, the incidence rates for other GLP-1RA drugs were similar to or higher than those for the placebo.

## 4 Discussion

This study systematically quantitatively assessed the efficacy and safety characteristics of 12 marketed and experimental GLP-1RA drugs. The results indicate significant variability in the efficacy of these drugs in reducing body weight. For instance, Liraglutide(1.8–3 mg) showed relatively lower efficacy, with a weight reduction of only 4.03 kg over 52 weeks compared to the placebo, whereas Retatrutide(6.5 mg) exhibited the highest efficacy, achieving a weight reduction of 13.2 kg compared to the placebo over the same period. This variation may be associated with the specificity of the drugs for in receptor interactions. The study found that the efficacy of tri-agonists was superior to that of dual-agonists and mono-agonists, with weight reductions of 24.15 kg, 11.07 kg, and 7.03 kg at 52 weeks, respectively. As a GLP-1/GIP/GCG tri-agonist, Retatrutide's interaction among the three receptors significantly enhanced its weight reduction effect. In comparison, although the GLP-1/GIP and GLP-1/GCG dual-agonists also showed a trend toward increased efficacy, their improvement was not as pronounced as that of the tri-agonists. Currently, Retatrutide is the only tri-agonist that has completed Phase II clinical trials [22]. More data are required to determine whether the weight reduction effects of tri-agonists generally surpass those of dual- and mono-agonists. Additionally, even within the same category of GLP-1 mono-agonists or dual-agonists, there was considerable variability in weight reduction effects, which may be related to the different affinities of drugs for receptors [23,24]. For example, among GLP-1 mono-agonists, Liraglutide had a relatively lower weight reduction effect (4.03 kg), whereas Orforglipron had the highest effect (8.66 kg). Among the GLP-1 dual-agonists, Cotadutide had the lowest weight reduction effect (4.73 kg), while BI 456906 had the highest (12.8 kg). Thus, apart from modifying the specificity of drugs to receptors, enhancing their affinity can significantly improve the weight reduction outcomes of GLP-1RA drugs.

This study found significant variations in the reported onset time of GLP-1RA drugs. For instance, Orforglipron demonstrated the fastest onset (6.4 weeks); conversely, Tirzepatide had the slowest onset (19.5 weeks), taking 46 weeks to reach its efficacy plateau. This indicates that when evaluating the efficacy of different GLP-1RA drugs, varying treatment durations should be employed to fully reflect the therapeutic potential of each drug. The duration of the clinical trials included in this study ranged from 6 to 104 weeks, with a median treatment duration of 26 weeks. The results suggested that for certain drugs, such as Tirzepatide, BI 456906, Semaglutide, and Mazdutide, a 26-week course only achieved 46.4 % to 69.4 % of their maximum effect. Overall, a 52-week treatment duration generally allows all the GLP-1RA drugs currently studied to reach their efficacy plateaus.

This study established the dose-response relationships for six GLP-1RA drugs, discovering that the doses required to reach 80 % of their maximum efficacy for Cotadutide, Danuglipron, JNJ-64565111, Retatrutide, Orforglipron, and injectable Semaglutide are 1.04 mg, 477 mg, 29.3 mg, 23 mg, 59.5 mg, and 3.07 mg, respectively. In current clinical trials, the maximum administered doses of these drugs are 0.6 mg, 200 mg, 10 mg, 12 mg, 45 mg, and 2.4 mg, corresponding to 70.8 %, 66.4 %, 65.8 %, 60.7 %, 75.2 %, and 77.3 % of their respective Emax values. These results suggest that increasing the dose of JNJ-64565111, while ensuring safety, could significantly enhance its weight reduction effects. However, the current dose of injectable Semaglutide (2.4 mg) is close to its efficacy plateau and further dose increases are unlikely to significantly improve its effectiveness. Additionally, no dose-response relationship was observed within the studied dosage range for the other six GLP-1RA drugs, such as Liraglutide (1.2–3 mg), where no significant change in weight reduction effect was noted within this range, indicating that the dosages of these drugs have reached their efficacy plateaus.

This study demonstrated that age significantly influences the weight-reduction effects of GLP-1RA drugs, with older individuals experiencing less weight reduction. For example, with Liraglutide, participants aged 30 years reduced their weight by 10.1 kg over 52 weeks, whereas those aged 60 years showed a reduction of 3.31 kg. Possible reasons for this phenomenon include a decline in basal metabolic rate with age, reduced muscle mass leading to decreased energy expenditure, and hormonal changes that affect lipid breakdown and energy utilization [25,26]. Therefore, when assessing the efficacy of GLP-1RA drugs, the heterogeneity in participants'ages must be considered. Additionally, age should be considered as a crucial factor for randomization and balance in the design of clinical trials for weight reduction medications.

Previous studies have shown that the efficacy of weight reduction medications is influenced by baseline weight, with individuals having higher baseline BMI experiencing larger reductions in weight [27]. However, in the covariate analysis of this study, no significant effects of baseline weight, baseline BMI, or proportion of males on the weight-reducing impact of GLP-1RA drugs were found. The participants included in this study had an average baseline weight of 72.2–121 kg and an average baseline BMI of 24.1–45.1 kg/m^2^, with the male proportion ranging from 19.1 % to 100 %. These findings suggest that within the above ranges, the weight reduction effects of GLP-1RA drugs are independent of baseline weight, BMI, and gender ratio. This may be related to the mechanism of action of GLP-1RA drugs. These findings provide a reference for developing inclusion and exclusion criteria for future clinical trials on GLP-1RA drugs.

The safety of weight reduction medications has long been a concern, with numerous drugs being withdrawn from the market owing to safety issues [28,29]. Participants experiencing adverse reactions or poor effectiveness during the use of weight reduction medications may opt to discontinue the drug. Thus, dropout rates can reflect the safety and efficacy of medications to some extent. This study found that the dropout rates for injectable Semaglutide, Liraglutide, Mazdutide, Retatrutide and Tirzepatide were significantly lower than those for the placebo, suggesting that these drugs have a favorable risk-benefit ratio. Conversely, the dropout rates for Orforglipron, oral Semaglutide, Danuglipron, BI 456906, Cotadutide and JNJ-64565111 were higher than those for placebo, which may be associated with the efficacy and incidence of adverse reactions such as constipation, nausea, diarrhea and vomiting.

In clinical trials, common adverse reactions to GLP-1 class drugs include gastrointestinal symptoms such as nausea, vomiting, diarrhea, and constipation [30,31]. Among the 12 GLP-1RA drugs studied, Orforglipron exhibited the highest incidence of nausea, constipation and diarrhea, while JNJ-65465111 exhibited the highest incidence of vomiting. These data can assist clinicians in selecting appropriate medications based on specific adverse reaction profiles of patients. Additionally, large-scale real-world studies indicate that GLP-1 class drugs may cause severe adverse reactions, such as acute pancreatitis, cholelithiasis, and acute kidney injury [[32], [33], [34], [35]]. However, owing to the limited sample sizes of clinical trials, the low incidence of these severe adverse reactions makes them difficult to detect in small-scale studies. Particularly, the safety profiles of GLP-1RA drugs that are still under development require further investigation with larger samples.

## 5 Limitations

This study has some limitations. First, some GLP-1RA drugs are still under development, and the related clinical trial data are limited, which may affect the robustness of the pharmacodynamic models. This is particularly true for drugs with few reported efficacy data points, making it challenging to accurately estimate their ET50 values. To address this, we employed a shared-parameter approach combined with a single-arm meta-analysis and Bayesian feedback to estimate these values. However, this method may introduce bias. Second, the data used in this study were derived from literature summaries rather than individual patient data, limiting our ability to access complete patient information and thus affecting the accuracy of our analysis of the factors influencing efficacy. Third, this study's comparison of the efficacy and safety of various GLP-1RA was not based on direct head-to-head trials, and some GLP-1RA drugs are still under development. Therefore, the final approved dosages or titration schedules for these medications may be subject to changes, which requires a cautious interpretation of the current findings. Finally, as the study included only English-language publications, there may have been a publication bias.

## 6 Conclusions

This study developed pharmacodynamic models for 12 GLP-1RA drugs and conducted quantitative analyses of their time-course relationships, dose-response relationships, factors influencing efficacy, dropout rates, and adverse events. These findings provide crucial quantitative data for evaluating new drugs.

● This study identified age as a significant factor affecting the weight reduction efficacy of these drugs.
● The efficacy of tri-agonists was significantly superior to that of dual- and mono-agonists.
● Common adverse events of GLP-1RAs, include nausea, vomiting, diarrhea, and constipation, with a significantly higher incidence of nausea than that of placebo.

## Contributors

Haoyang Guo and Juan Yang wrote the manuscript; Qingshan Zheng and Lujin Li designed the research; Haoyang Guo, Jihan Huang, Ling Xu and Yinghua Lv performed the research; Haoyang Guo, Yexuan Wang, Jiyuan Ren and Yulin Feng analyzed the data.

## Data sharing statement

Data described in the manuscript, code book, and analytic code will be made available upon request pending application and approval.

## Declaration of artificial intelligence

All conceptualizations, methodologies, analyses, and content development were conducted manually by the authors without the use of any artificial intelligence tools or systems.

## Funding

This work received financial support from three sources: China national key research and development program (2022YFC3502000), National Natural Science Funds of China (82174229), and Shanghai S&T Innovation Plan (17401970900).

## Declaration of competing interest

The authors declare that they have no known competing financial interests or personal relationships that could have appeared to influence the work reported in this paper.
