# Supplementary material for: Comparative efficacy and safety of GLP-1 receptor agonists for weight reduction: A model-based meta-analysis of placebo-controlled trials
Source: Obes Pillars. 2025 Jan 30;13:100162. doi: 10.1016/j.obpill.2025.100162 (PMC11840199; doi:10.1016/j.obpill.2025.100162)
Supplement: Multimedia component 2 [file mmc2.docx]

Supplementary

[Table S1. Literature search strategy 1](#_Toc174881559)

[Figure S1. Flow diagram of study selection for inclusion in the analysis 2](#_Toc174881560)

[Table S2. The list of the included studies 3](#_Toc174881561)

[Table S3. The details of the included studies 8](#_Toc174881562)

[Table S4. Characteristics of the included studies, median (min~max) 14](#_Toc174881563)

[Table S5. Risk assessment of the included studies 15](#_Toc174881564)

[Figure S2. The summary risk of literature assessment 18](#_Toc174881565)

[Table S6. Parameter estimates of the final model 19](#_Toc174881566)

[Figure S3. Onset times of different GLP-1 receptor agonists 20](#_Toc174881567)

[Figure S4. The goodness-of-fit plots of the final model 21](#_Toc174881568)

[Figure S5. Correlation plot of model parameters and covariates 22](#_Toc174881569)

[Methods 1. Model building 23](#_Toc174881570)

[Methods 2. Model evaluation 25](#_Toc174881571)

# Table S1. Literature search strategy

| No. | Query | Results |
| --- | --- | --- |
| PubMed | |  |
| #1 | Obesity[Mesh] OR Overweight[Mesh] OR Weight Loss[Mesh] | 303377 |
| #2 | Obesity[Text Word] OR Overweight[Text Word] OR Weight Loss[Text Word] | 517866 |
| #3 | #1 OR #2 | 524624 |
| #4 | GLP-1[Text Word] | 15367 |
| #5 | GLP-1[Mesh] | 11351 |
| #6 | #4 OR #5 | 18741 |
| #7 | #3 AND #6 Clinical Trial, Humans | 739 |
| Embase | |  |
| #1 | Obesity/exp OR Overweight/exp OR Weight Loss/exp OR Obesity:ti,ab,kw OR Overweight:ti,ab,kw OR Weight Loss:ti,ab,kw | 990686 |
| #2 | GLP-1/exp OR GLP-1:ti,ab,kw | 34700 |
| #3 | #1 AND #2 | 12461 |
| #4 | #3 AND clinical trial/de AND human/de | 1050 |

# Figure S1. Flow diagram of study selection for inclusion in the analysis


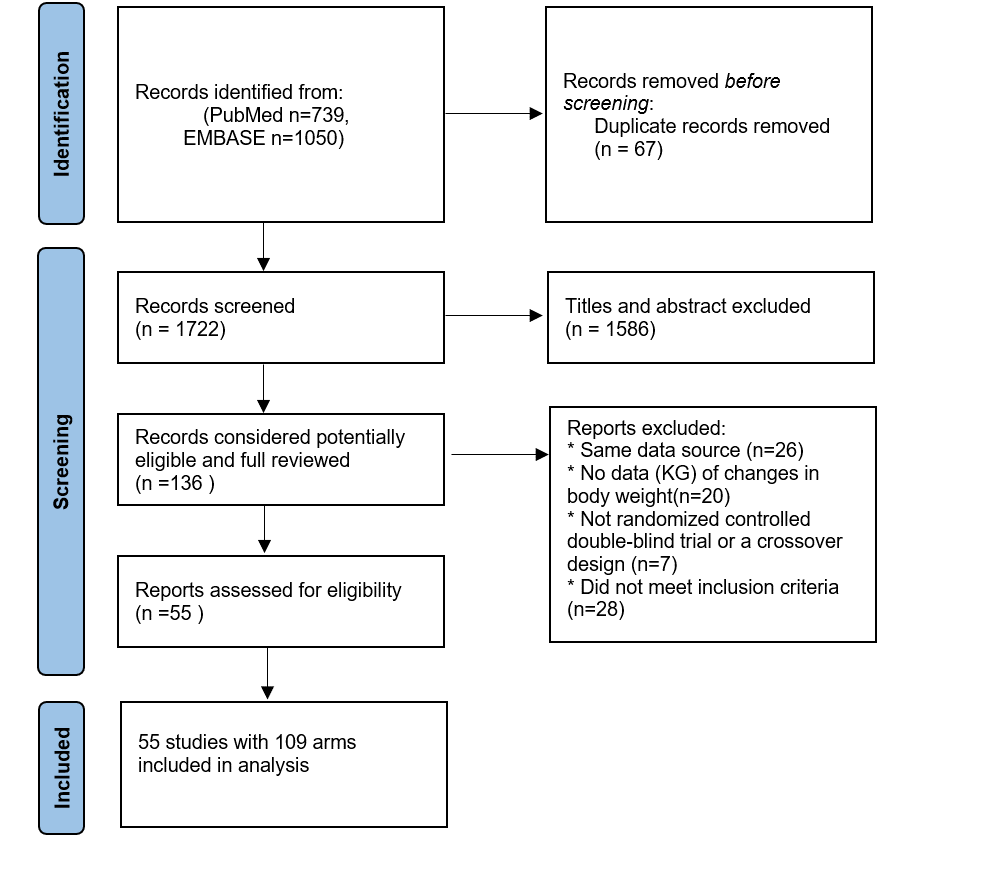


# Table S2. The list of the included studies

| ID | Author | Publication  year | Article title |
| --- | --- | --- | --- |
| 1 | Arrubla, J. | 2022 | A randomized Phase I study of the safety, tolerability, pharmacokinetics and pharmacodynamics of BI 456906, a dual glucagon receptor/glucagon-like peptide-1 receptor agonist, in healthy Japanese men with overweight/obesity |
| 2 | Jungnik, A. | 2022 | Phase I studies of the safety, tolerability, pharmacokinetics and pharmacodynamics of the dual glucagon receptor/ glucagon-like peptide-1 receptor agonist BI 456906 |
| 3 | Ambery, P. | 2018 | MEDI0382, a GLP-1 and glucagon receptor dual agonist, in obese or overweight patients with type 2 diabetes: a randomised, controlled, double-blind, ascending dose and phase 2a study |
| 4 | Asano, M. | 2021 | Pharmacokinetics, safety, tolerability and efficacy of cotadutide, a glucagon-like peptide-1 and glucagon receptor dual agonist, in phase 1 and 2 trials in overweight or obese participants of Asian descent with or without type 2 diabetes |
| 5 | Asano, M. | 2022 | Safety/tolerability, efficacy and pharmacokinetics of 600-μg cotadutide in Japanese type 2 diabetes patients with a body mass index of 25 kg/m2 or higher: A phase I, randomized, double-blind, placebo-controlled study |
| 6 | Nahra, R. | 2021 | Effects of cotadutide on metabolic and hepatic parameters in adults with overweight or obesity and type 2 diabetes: A 54-week randomized phase 2b study |
| 7 | Saxena, A. R. | 2023 | Efficacy and safety of oral small molecule glucagon-like peptide 1 receptor agonist danuglipron for glycemic control among patients with type 2 diabetes A randomized clinical trial |
| 8 | Saxena, A. R. | 2023 | Tolerability, safety and pharmacodynamics of oral, small-molecule glucagon-like peptide-1 receptor agonist danuglipron for type 2 diabetes: A 12-week, randomized, placebo-controlled, Phase 2 study comparing different dose-escalation schemes |
| 9 | Camastra, S. | 2017 | Effect of exenatide on postprandial glucose fluxes, lipolysis, and ß-cell function in non-diabetic, morbidly obese patients |
| 10 | Dutour, A. | 2016 | Exenatide decreases liver fat content and epicardial adipose tissue in patients with obesity and type 2 diabetes: a prospective randomized clinical trial using magnetic resonance imaging and spectroscopy. |
| 11 | Siskind, D. J. | 2018 | Treatment of clozapine-associated obesity and diabetes with exenatide in adults with schizophrenia: A randomized controlled trial (CODEX) |
| 12 | Alba, M. | 2021 | Efficacy and safety of glucagon-like peptide-1/glucagon receptor co-agonist JNJ-64565111 in individuals with obesity without type 2 diabetes mellitus: A randomized dose-ranging study. |
| 13 | Di Prospero, N. A. | 2021 | Efficacy and safety of glucagon-like peptide-1/glucagon receptor co-agonist JNJ-64565111 in individuals with type 2 diabetes mellitus and obesity: A randomized dose-ranging study. |
| 14 | Astrup, A. | 2012 | Safety, tolerability and sustained weight loss over 2 years with the once-daily human GLP-1 analog, liraglutide. |
| 15 | Astrup, A. | 2009 | Effects of liraglutide in the treatment of obesity: a randomised, double-blind, placebo-controlled study. |
| 16 | Blackman, A. | 2016 | Effect of liraglutide 3.0 mg in individuals with obesity and moderate or severe obstructive sleep apnea: the SCALE Sleep Apnea randomized clinical trial. |
| 17 | Davies, M. J. | 2015 | Efficacy of liraglutide for weight loss among patients with type 2 diabetes: the SCALE diabetes randomized clinical trial. |
| 18 | Dejgaard, T. F. | 2016 | Efficacy and safety of liraglutide for overweight adult patients with type 1 diabetes and insufficient glycaemic control (Lira-1): a randomised, double-blind, placebo-controlled trial. |
| 19 | Dejgaard, T. F. | 2020 | Liraglutide reduces hyperglycaemia and body weight in overweight, dysregulated insulin-pump-treated patients with type 1 diabetes: The Lira Pump trial-a randomized, double-blinded, placebo-controlled trial. |
| 20 | Ghanim, H. | 2020 | Liraglutide treatment in overweight and obese patients with type 1 diabetes: A 26-week randomized controlled trial; mechanisms of weight loss. |
| 21 | Halawi, H. | 2017 | Effects of liraglutide on weight, satiation, and gastric functions in obesity: a randomised, placebo-controlled pilot trial. |
| 22 | Kim, S. H. | 2013 | Benefits of liraglutide treatment in overweight and obese older individuals with prediabetes. |
| 23 | Larsen, J. R. | 2017 | Effect of liraglutide treatment on prediabetes and overweight or obesity in clozapine- or olanzapine-treated patients with schizophrenia spectrum disorder: A randomized clinical trial. |
| 24 | Lind, M. | 2015 | Liraglutide in people treated for type 2 diabetes with multiple daily insulin injections: randomised clinical trial (MDI Liraglutide trial). |
| 25 | O'Neil, P. M. | 2018 | Efficacy and safety of semaglutide compared with liraglutide and placebo for weight loss in patients with obesity: a randomised, double-blind, placebo and active controlled, dose-ranging, phase 2 trial. |
| 26 | Pi-Sunyer, X. | 2015 | A randomized, controlled trial of 3.0 mg of liraglutide in weight management. |
| 27 | Robert, S. A. | 2015 | Improvement in binge eating in non-diabetic obese individuals after 3 months of treatment with liraglutide - A pilot study. |
| 28 | Schmidt, S. | 2022 | Liraglutide changes body composition and lowers added sugar intake in overweight persons with insulin pump-treated type 1 diabetes. |
| 29 | Smits, M. M. | 2016 | Twelve week liraglutide or sitagliptin does not affect hepatic fat in type 2 diabetes: a randomised placebo-controlled trial. |
| 30 | van Eyk, H. J. | 2020 | Liraglutide decreases energy expenditure and does not affect the fat fraction of supraclavicular brown adipose tissue in patients with type 2 diabetes. |
| 31 | Whicher, C. A. | 2021 | The use of liraglutide 3.0 mg daily in the management of overweight and obesity in people with schizophrenia, schizoaffective disorder and first episode psychosis: Results of a pilot randomized, double-blind, placebo-controlled trial. |
| 32 | Jastreboff, A. M. | 2023 | Triple-hormone-receptor agonist retatrutide for obesity - A phase 2 trial. |
| 33 | Rosenstock, J. | 2023 | Retatrutide, a GIP, GLP-1 and glucagon receptor agonist, for people with type 2 diabetes: a randomised, double-blind, placebo and active-controlled, parallel-group, phase 2 trial conducted in the USA. |
| 34 | Frias, J. P. | 2023 | Efficacy and safety of oral orforglipron in patients with type 2 diabetes: a multicentre, randomised, dose-response, phase 2 study. |
| 35 | Wharton, S. | 2023 | Daily oral GLP-1 receptor agonist orforglipron for adults with obesity. |
| 36 | Blundell, J. | 2017 | Effects of once-weekly semaglutide on appetite, energy intake, control of eating, food preference and body weight in subjects with obesity. |
| 37 | Davies, M. | 2021 | Semaglutide 2·4 mg once a week in adults with overweight or obesity, and type 2 diabetes (STEP 2): a randomised, double-blind, double-dummy, placebo-controlled, phase 3 trial. |
| 38 | Mu, Y. | 2023 | Efficacy and safety of once weekly semaglutide 2.4 mg for weight management in a predominantly Asian population with overweight or obesity in the STEP 7 randomised clinical trial. |
| 39 | Wilding, J. P. H. | 2021 | Once-weekly semaglutide in adults with overweight or obesity. |
| 40 | Marso, S. P. | 2016 | Semaglutide and cardiovascular outcomes in patients with type 2 diabetes. |
| 41 | Garvey, W. T. | 2022 | Two-year effects of semaglutide in adults with overweight or obesity: the STEP 5 trial. |
| 42 | Kadowaki, T. | 2022 | Semaglutide once a week in adults with overweight or obesity, with or without type 2 diabetes in an east Asian population (STEP 6): a randomised, double-blind, double-dummy, placebo-controlled, phase 3a trial |
| 43 | Sorli, C. | 2017 | Efficacy and safety of once-weekly semaglutide monotherapy versus placebo in patients with type 2 diabetes (SUSTAIN 1): a double-blind, randomised, placebo-controlled, parallel-group, multinational, multicentre phase 3a trial. |
| 44 | Zinman, B. | 2019 | Semaglutide once weekly as add-on to SGLT-2 inhibitor therapy in type 2 diabetes (SUSTAIN 9): a randomised, placebo-controlled trial. |
| 45 | Wharton, S. | 2023 | Two-year effect of semaglutide 2.4 mg on control of eating in adults with overweight/obesity: STEP 5. |
| 46 | Lingvay, I. | 2018 | A 26-week randomized controlled trial of semaglutide once daily versus liraglutide and placebo in patients with type 2 diabetes suboptimally controlled on diet and exercise with or without metformin. |
| 47 | Davies, M. | 2017 | Effect of oral semaglutide compared with placebo and subcutaneous semaglutide on glycemic control in patients with type 2 diabetes: A randomized clinical trial. |
| 48 | Pratley, R. | 2019 | Oral semaglutide versus subcutaneous liraglutide and placebo in type 2 diabetes (PIONEER 4): a randomised, double-blind, phase 3a trial." |
| 49 | Dahl, D. | 2022 | Effect of subcutaneous tirzepatide vs placebo added to titrated insulin glargine on glycemic control in patients with type 2 diabetes: the SURPASS-5 randomized clinical trial. |
| 50 | Garvey, W. T. | 2023 | Tirzepatide once weekly for the treatment of obesity in people with type 2 diabetes (SURMOUNT-2): a double-blind, randomised, multicentre, placebo-controlled, phase 3 trial. |
| 51 | Jastreboff, A. M. | 2022 | Tirzepatide Once Weekly for the Treatment of Obesity. |
| 52 | Rosenstock, J. | 2021 | Efficacy and safety of a novel dual GIP and GLP-1 receptor agonist tirzepatide in patients with type 2 diabetes (SURPASS-1): a double-blind, randomised, phase 3 trial. |
| 53 | Ji, L. | 2021 | IBI362 (LY3305677), a weekly-dose GLP-1 and glucagon receptor dual agonist, in Chinese adults with overweight or obesity: A randomised, placebo-controlled, multiple ascending dose phase 1b study. |
| 54 | Ji, L. | 2023 | A phase 2 randomised controlled trial of mazdutide in Chinese overweight adults or adults with obesity. |
| 55 | Urva, S. | 2022 | LY3437943, a novel triple GIP, GLP-1, and glucagon receptor agonist in people with type 2 diabetes: a phase 1b, multicentre, double-blind, placebo-controlled, randomised, multiple-ascending dose trial. |

# Table S3. The details of the included studies

| ID | Study | Intervention | Sample size of treatment arm | Treatment  duration(week) | Dose(mg) | BMI | Weight(kg) | Male(%) | Age(year) | Dropout  rate, % |
| --- | --- | --- | --- | --- | --- | --- | --- | --- | --- | --- |
| 101 | Arrubla, J.2022 | BI 456906 | 9 | 16 | 1.8 | 25.6 | 73.8 | 100 | 37 | 0 |
| 102 | Arrubla, J.2022 | BI 456906 | 9 | 16 | 4.8 | 24.1 | 72.2 | 100 | 31.9 | 0 |
| 103 | Arrubla, J.2022 | BI 456906 | 9 | 16 | 2.4 | 25.1 | 77.8 | 100 | 31.6 | 0 |
| 201 | Jungnik, A.2022 | BI 456906 | 13 | 16 | 2.4 | 29.6 | 87.7 | 69.2 | 45.0 | 38 |
| 202 | Jungnik, A.2022 | BI 456906 | 11 | 16 | 4.8 | 30.5 | 94.2 | 63.3 | 48.9 | 18 |
| 203 | Jungnik, A.2022 | BI 456906 | 9 | 16 | 2.4 | 31.7 | 95.8 | 75.0 | 42.2 | 11 |
| 301 | Ambery, P.2018 | Cotadutide | 25 | 6 | 0.2 | 32.0 | 95.9 | 52.0 | 56.0 | 12 |
| 401 | Asano, M.2021 | Cotadutide | 15 | 7 | 0.1 | 28.8 | 77.1 | 53.0 | 56.7 | 0 |
| 402 | Asano, M.2021 | Cotadutide | 15 | 7 | 0.2 | 26.4 | 73.1 | 87.0 | 58.7 | 33 |
| 403 | Asano, M.2021 | Cotadutide | 15 | 7 | 0.3 | 27.0 | 76.7 | 87.0 | 57.5 | 27 |
| 501 | Asano, M.2022 | Cotadutide | 12 | 12 | 0.6 | 27.2 | 77.1 | 58.3 | 58.5 | 33 |
| 601 | Nahra, R.2021 | Cotadutide | 100 | 54 | 0.1 | 35.0 | 99.0 | 43.0 | 57.6 | 4 |
| 602 | Nahra, R.2021 | Cotadutide | 256 | 54 | 0.2 | 34.9 | 98.1 | 43.0 | 57.3 | 5 |
| 603 | Nahra, R.2021 | Cotadutide | 256 | 54 | 0.3 | 35.2 | 100.8 | 50.0 | 56.3 | 4 |
| 701 | Saxena, A. R.2023 | Danuglipron | 68 | 16 | 10 | 33.0 | 92.2 | 51.0 | 58.1 | 7 |
| 702 | Saxena, A. R.2023 | Danuglipron | 71 | 16 | 40 | 32.3 | 90.0 | 48.0 | 58.8 | 20 |
| 703 | Saxena, A. R.2023 | Danuglipron | 67 | 16 | 80 | 32.9 | 91.6 | 52.0 | 58.4 | 30 |
| 704 | Saxena, A. R.2023 | Danuglipron | 71 | 16 | 120 | 33.3 | 93.0 | 48.0 | 58.8 | 46 |
| 801 | Saxena, A. R.2023 | Danuglipron | 22 | 12 | 120 | 33.9 | 95.2 | 54.5 | 57.2 | 32 |
| 802 | Saxena, A. R.2023 | Danuglipron | 21 | 12 | 200 | 31.5 | 86.4 | 52.4 | 59.0 | 38 |
| 901 | Camastra, S.2017 | Exenatide | 15 | 12 | 0.01 | 45.1 | 121.0 | NA | 47 | 0 |
| 1001 | Dutour, A.2016 | Exenatide | 22 | 26 | 0.01 | 37.2 | 104.0 | 59.0 | 51 | 14 |
| 1101 | Siskind, D. J.2018 | Exenatide | 14 | 24 | 2.0 | 35.6 | 108.0 | 61.1 | NA | 0 |
| 1201 | Alba, M.2021 | JNJ-64565111 | 47 | 12 | 5.0 | 40.4 | 111.4 | 33.3 | 55.1 | 32 |
| 1202 | Alba, M.2021 | JNJ-64565111 | 48 | 12 | 7.4 | 40.4 | 113.4 | 42.9 | 57.8 | 40 |
| 1203 | Alba, M.2021 | JNJ-64565111 | 48 | 12 | 10 | 41.0 | 116.4 | 42.9 | 56.2 | 31 |
| 1301 | Di Prospero, N. A.2021 | JNJ-64565111 | 59 | 26 | 5.0 | 40.3 | 112.4 | 20.3 | 47.3 | 27 |
| 1302 | Di Prospero, N. A.2021 | JNJ-64565111 | 118 | 26 | 7.4 | 40.0 | 112.0 | 27.1 | 46.2 | 31 |
| 1303 | Di Prospero, N. A.2021 | JNJ-64565111 | 118 | 26 | 10 | 40.5 | 113.7 | 27.1 | 46.2 | 39 |
| 1401 | Astrup, A.2012 | Liraglutide | 95 | 52 | 1.2 | 34.8 | 98.0 | 23.0 | 47.2 | 18 |
| 1402 | Astrup, A.2012 | Liraglutide | 90 | 52 | 1.8 | 35.0 | 99.3 | 24.0 | 45.5 | 17 |
| 1403 | Astrup, A.2012 | Liraglutide | 93 | 52 | 2.4 | 35.0 | 99.7 | 24.0 | 45.0 | 9 |
| 1404 | Astrup, A.2012 | Liraglutide | 93 | 52 | 3.0 | 34.8 | 98.9 | 25.0 | 45.9 | 11 |
| 1501 | Astrup, A.2009 | Liraglutide | 95 | 20 | 1.2 | 34.8 | 96.2 | 23.0 | 47.2 | 11 |
| 1502 | Astrup, A.2009 | Liraglutide | 90 | 20 | 1.8 | 35.0 | 98.0 | 24.0 | 45.5 | 18 |
| 1503 | Astrup, A.2009 | Liraglutide | 93 | 20 | 2.4 | 35.0 | 98.4 | 24.0 | 45.0 | 22 |
| 1504 | Astrup, A.2009 | Liraglutide | 93 | 20 | 3.0 | 34.8 | 97.6 | 25.0 | 45.9 | 12 |
| 1601 | Blackman, A.2016 | Liraglutide | 180 | 32 | 3.0 | 38.9 | 116.5 | 71.7 | 48.6 | 26 |
| 1701 | Davies, M. J.2015 | Liraglutide | 204 | 56 | 1.8 | 37.0 | 105.8 | 51.2 | 54.9 | 5 |
| 1702 | Davies, M. J.2015 | Liraglutide | 411 | 56 | 3.0 | 37.1 | 105.7 | 52.0 | 55.0 | 3 |
| 1801 | Dejgaard, T. F.2016 | Liraglutide | 50 | 24 | 1.8 | 30.3 | 93.4 | 60.0 | 47.0 | 8 |
| 1901 | Dejgaard, T. F.2020 | Liraglutide | 22 | 26 | 1.8 | 30.0 | 85.0 | 32.0 | 50.0 | 9 |
| 2001 | Ghanim, H.2020 | Liraglutide | 42 | 26 | 1.8 | 29.6 | 94.2 | 41.0 | 45.0 | 12 |
| 2101 | Halawi, H.2017 | Liraglutide | 19 | 16 | 3.0 | 37.2 | 103.7 | NA | 42.0 | 11 |
| 2201 | Kim, S. H.2013 | Liraglutide | 24 | 14 | 1.8 | 31.9 | 88.4 | 33.0 | 58.0 | 33 |
| 2301 | Larsen, J. R.2017 | Liraglutide | 47 | 16 | 1.8 | 33.7 | 103.3 | 63.8 | 42.1 | 11 |
| 2401 | Lind, M.2015 | Liraglutide | 63 | 24 | 1.8 | NA | NA | 63.5 | 63.8 | 2 |
| 2501 | O'Neil, P. M.2018 | Liraglutide | 103 | 52 | 3.0 | 38.6 | 108.7 | 35.0 | 49.0 | 17 |
| 2601 | Pi-Sunyer, X.2015 | Liraglutide | 2487 | 52 | 3.0 | 38.3 | 106.2 | 21.3 | 45.2 | 28 |
| 2701 | Robert, S. A.2015 | Liraglutide | 21 | 12 | 1.8 | 36.2 | 94.5 | NA | NA | 0 |
| 2801 | Schmidt, S.2022 | Liraglutide | 22 | 26 | 1.8 | 30.2 | 85.9 | 68.2 | 54.0 | 14 |
| 2901 | Smits, M. M.2016 | Liraglutide | 17 | 12 | 1.8 | 32.8 | 103.2 | 70.6 | 60.8 | 0 |
| 3001 | van Eyk, H. J.2020 | Liraglutide | 23 | 26 | 1.8 | 32.6 | 98.4 | 61.0 | 59.9 | 9 |
| 3101 | Whicher, C. A.2021 | Liraglutide | 24 | 26 | 3.0 | 37.5 | 111.4 | 62.0 | 42.7 | 21 |
| 3201 | Jastreboff, A. M.2023 | Retatrutide | 69 | 48 | 1.0 | 37.5 | 106.4 | 52.0 | 50.6 | 13 |
| 3202 | Jastreboff, A. M.2023 | Retatrutide | 62 | 48 | 12 | 37.4 | 108.0 | 52.0 | 45.8 | 13 |
| 3301 | Rosenstock, J.2023 | Retatrutide | 47 | 36 | 0.5 | 34.7 | 96.7 | 51.0 | 57.2 | 13 |
| 3302 | Rosenstock, J.2023 | Retatrutide | 46 | 36 | 12 | 35.5 | 99.9 | 43.0 | 54.9 | 24 |
| 3401 | Frias, J. P.2023 | Orforglipron | 51 | 26 | 3.0 | 35.3 | 99.3 | 51.0 | 59.0 | 8 |
| 3402 | Frias, J. P.2023 | Orforglipron | 56 | 26 | 12 | 34.8 | 99.3 | 64.0 | 57.4 | 11 |
| 3403 | Frias, J. P.2023 | Orforglipron | 47 | 26 | 24 | 34.1 | 98.5 | 64.0 | 60.5 | 11 |
| 3404 | Frias, J. P.2023 | Orforglipron | 61 | 26 | 36 | 34.4 | 98.9 | 59.0 | 59.7 | 10 |
| 3405 | Frias, J. P.2023 | Orforglipron | 63 | 26 | 45 | 36.4 | 104.6 | 63.0 | 58.5 | 8 |
| 3501 | Wharton, S.2023 | Orforglipron | 50 | 36 | 12 | 37.7 | 107.5 | 38.0 | 49.8 | 26 |
| 3502 | Wharton, S.2023 | Orforglipron | 53 | 36 | 24 | 38.1 | 112.1 | 47.0 | 57.0 | 26 |
| 3601 | Blundell, J.2017 | Semaglutide(INJ.) | 14 | 12 | 1.0 | 33.8 | 101.3 | 66.7 | 42.0 | 14 |
| 3701 | Davies, M.2021 | Semaglutide(INJ.) | 403 | 68 | 1.0 | 35.3 | 99.0 | 49.6 | 56.0 | 3 |
| 3702 | Davies, M.2021 | Semaglutide(INJ.) | 404 | 68 | 2.4 | 35.9 | 99.9 | 54.8 | 55.0 | 3 |
| 3801 | Mu, Y.2023 | Semaglutide(INJ.) | 407 | 68 | 2.4 | 38.1 | 106.9 | 22.6 | 46.0 | 7 |
| 3901 | Wilding, J. P. H.2021 | Semaglutide(INJ.) | 1306 | 68 | 2.4 | 37.8 | 105.4 | 36.9 | 46.0 | 5 |
| 4001 | Marso, S. P.2016 | Semaglutide(INJ.) | 826 | 104 | 0.5 | NA | 92.0 | 59.9 | 64.6 | 3 |
| 4002 | Marso, S. P.2016 | Semaglutide(INJ.) | 822 | 104 | 1.0 | NA | 92.0 | 63.0 | 64.7 | 2 |
| 4101 | Garvey, W. T.2022 | Semaglutide(INJ.) | 152 | 104 | 2.4 | 38.6 | 105.6 | 19.1 | 47.3 | 3 |
| 4201 | Kadowaki, T.2022 | Semaglutide(INJ.) | 101 | 68 | 1.7 | 31.6 | 86.1 | 63.0 | 51.0 | 2 |
| 4202 | Kadowaki, T.2022 | Semaglutide(INJ.) | 199 | 68 | 2.4 | 32.0 | 86.9 | 57.0 | 52.0 | 2 |
| 4301 | Sorli, C.2017 | Semaglutide(INJ.) | 129 | 30 | 0.5 | 32.5 | 91.9 | 46.9 | 54.6 | 13 |
| 4302 | Sorli, C.2017 | Semaglutide(INJ.) | 130 | 30 | 1.0 | 33.9 | 91.9 | 61.5 | 52.7 | 12 |
| 4401 | Zinman, B.2019 | Semaglutide(INJ.) | 151 | 30 | 1.0 | 31.1 | 91.7 | 58.9 | 57.5 | 3 |
| 4501 | Wharton, S.2023 | Semaglutide(INJ.) | 88 | 104 | 2.4 | 39.3 | 107.0 | 19.3 | 47.3 | 3 |
| 4601 | Lingvay, I.2018 | Semaglutide(INJ.) | 64 | 26 | 0.05 | NA | 93.4 | 51.6 | 57.5 | 14 |
| 4602 | Lingvay, I.2018 | Semaglutide(INJ.) | 63 | 26 | 0.1 | NA | 92.4 | 60.0 | 57.5 | 16 |
| 4603 | Lingvay, I.2018 | Semaglutide(INJ.) | 65 | 26 | 0.2 | NA | 98.1 | 58.9 | 58.4 | 15 |
| 4604 | Lingvay, I.2018 | Semaglutide(INJ.) | 63 | 26 | 0.3 | NA | 94.8 | 53.8 | 54.8 | 35 |
| 4701 | Davies, M.2017 | Semaglutide(P.O) | 70 | 26 | 2.5 | 31.7 | 92.3 | 45.0 | 56.7 | 33 |
| 4702 | Davies, M.2017 | Semaglutide(P.O) | 70 | 26 | 5.0 | 34.6 | 92.3 | 47.0 | 55.7 | 23 |
| 4703 | Davies, M.2017 | Semaglutide(P.O) | 69 | 26 | 10 | 31.9 | 92.3 | 43.0 | 56.5 | 6 |
| 4704 | Davies, M.2017 | Semaglutide(P.O) | 70 | 26 | 20 | 32.0 | 92.3 | 44.0 | 58.3 | 7 |
| 4705 | Davies, M.2017 | Semaglutide(P.O) | 71 | 26 | 40 | 31.1 | 92.3 | 43.0 | 56.5 | 8 |
| 4706 | Davies, M.2017 | Semaglutide(P.O) | 69 | 26 | 1.0 | 30.7 | 92.3 | 48.0 | 56.8 | 7 |
| 4801 | Pratley, R.2019 | Semaglutide(P.O) | 285 | 52 | 14 | 32.5 | 108.3 | 52.0 | 56.0 | 3 |
| 4901 | Dahl, D.2022 | Tirzepatide | 116 | 40 | 5.0 | 33.6 | 95.8 | 53.0 | 62.0 | 6 |
| 4902 | Dahl, D.2022 | Tirzepatide | 118 | 40 | 10 | 33.4 | 94.5 | 61.0 | 60.0 | 3 |
| 4903 | Dahl, D.2022 | Tirzepatide | 120 | 40 | 15 | 33.4 | 96.3 | 54.0 | 61.0 | 8 |
| 5001 | Garvey, W. T.2023 | Tirzepatide | 312 | 72 | 10 | 36.0 | 100.9 | 49.0 | 54.3 | 5 |
| 5002 | Garvey, W. T.2023 | Tirzepatide | 311 | 72 | 15 | 35.7 | 99.6 | 51.0 | 53.6 | 9 |
| 5101 | Jastreboff, A. M.2022 | Tirzepatide | 630 | 72 | 5.0 | 37.4 | 113.2 | 30.1 | 48.0 | 11 |
| 5102 | Jastreboff, A. M.2022 | Tirzepatide | 636 | 72 | 10 | 38.2 | 114.8 | 30.0 | 46.5 | 12 |
| 5103 | Jastreboff, A. M.2022 | Tirzepatide | 630 | 72 | 15 | 38.1 | 114.4 | 29.9 | 47.2 | 10 |
| 5201 | Rosenstock, J.2021 | Tirzepatide | 121 | 40 | 5.0 | 32.2 | 87.0 | 46.0 | 54.2 | 6 |
| 5202 | Rosenstock, J.2021 | Tirzepatide | 121 | 40 | 10 | 32.2 | 85.7 | 60.0 | 55.8 | 7 |
| 5203 | Rosenstock, J.2021 | Tirzepatide | 121 | 40 | 15 | 31.5 | 85.9 | 52.0 | 52.9 | 15 |
| 5301 | Ji, L.2021 | Mazdutide | 8 | 12 | 3.0 | 29.3 | 78.3 | 37.5 | 29.5 | 0 |
| 5302 | Ji, L.2021 | Mazdutide | 8 | 12 | 4.5 | 32.4 | 93.1 | 25.0 | 31.0 | 0 |
| 5303 | Ji, L.2021 | Mazdutide | 8 | 12 | 6.0 | 31.7 | 87.4 | 62.5 | 40.0 | 13 |
| 5401 | Ji, L.2023 | Mazdutide | 62 | 24 | 3.0 | 31.8 | 89.8 | 43.5 | 37.2 | 11 |
| 5402 | Ji, L.2023 | Mazdutide | 63 | 24 | 4.5 | 31.8 | 89.3 | 41.3 | 33.6 | 5 |
| 5403 | Ji, L.2023 | Mazdutide | 61 | 24 | 6.0 | 31.7 | 88.5 | 55.7 | 35.8 | 8 |
| 5501 | Urva, S.2022 | Mazdutide | 8 | 12 | 9.0 | 31.8 | 82.8 | 25.0 | 36.0 | 0 |
| 5502 | Urva, S.2022 | Mazdutide | 8 | 12 | 10 | 30.1 | 79.8 | 25.0 | 37.9 | 13 |

# Table S4. Characteristics of the included studies, median (min~max)

|  | **Number of arms** | **Total sample size** | **Age, years** | **Baseline of**  **weight, kg** | **BMI, kg/m^3^** | **Male,%** | **Dose, mg** |
| --- | --- | --- | --- | --- | --- | --- | --- |
| BI 456906 | 6 | 66 | 39.1(31.6~48.9) | 81.6(72.2~95.8) | 27.1(24.1~31.7) | 85.7(63.3~100.0) | 3.3(1.8~4.8) |
| Cotadutide | 8 | 694 | 57.8(56.0~58.7) | 83.1(73.1~100.8) | 29.4(26.4~35.2) | 59.3(43.0~87.0) | 0.4(0.1~0.6) |
| Danuglipron | 6 | 320 | 58.4(57.2~59.6) | 92.2(86.4~95.2) | 33.0(31.5~33.9) | 50.7(48.0~54.5) | 77.5(10.0~200.0) |
| Exenatide | 3 | 51 | 48.3(47.0~51.0) | 111.6(104.0~121.0) | 40.0(35.6~45.1) | 59.7(59.0~61.1) | 0.4(0.01~2.0) |
| JNJ-64565111 | 6 | 441 | 51.3(46.2~57.8) | 113.0(111.4~13.7) | 40.4(40.0~41.0) | 31.5(20.3~42.9) | 7.0(5.0~10.0) |
| Liraglutide | 25 | 4538 | 49.8(42.0~63.8) | 101.2(85.0~116.5) | 35.4(29.6~38.9) | 41.8(21.3~71.7) | 2.3(1.2~3) |
| Retatrutide | 4 | 224 | 51.1(45.8~57.2) | 103.8(96.7~108.0) | 36.6(34.7~37.5) | 51.4(43.0~52.0) | 4.9(0.5~12) |
| Orforglipron | 7 | 381 | 57.0(49.8~60.5) | 102.0(98.5~112.1) | 35.7(34.1~38.1) | 55.1(38.0~64.0) | 21.4(3.0~45.0) |
| Semaglutide(INJ.) | 18 | 5388 | 53.9(42.0~64.7) | 96.4(86.1~107.0) | 34.6(31.1~39.3) | 45.6(19.1~66.7) | 4.3(0.05~2.4) |
| Semaglutide(P.O) | 7 | 704 | 56.9(55.7~58.3) | 93.4(92.3~108.3) | 32.0(30.7~34.6) | 49.5(43.0~52.0) | 8.0(2.5~40.0) |
| Tirzepatide | 11 | 3236 | 53.6(46.5~62.0) | 101.1(85.7~114.8) | 35.2(31.5~38.2) | 45.3(29.9~61.0) | 10.2(5.0~15.0) |
| Mazdutide | 8 | 226 | 35.0(29.5~40.0) | 87.4(78.3~89.8) | 31.5(29.3~32.4) | 42.3(25.0~62.5) | 5.1(3.0~10.0) |

# Table S5. Risk assessment of the included studies

| No | Study | Randomization process | Deviations from intended interventions | Missing outcome data | Measurement of the outcome | Selection of the reported result | Overall bias | Weight |
| --- | --- | --- | --- | --- | --- | --- | --- | --- |
| 1 | Arrubla, J.2022 | Low | Some concerns | Low | Low | Low | Some concerns | 1 |
| 2 | Jungnik, A.2022 | Low | Some concerns | Low | Low | Low | Some concerns | 1 |
| 3 | Ambery, P.2018 | Low | Low | Low | Low | Low | Low | 1 |
| 4 | Asano, M.2021 | Low | Some concerns | Low | Low | Low | Some concerns | 1 |
| 5 | Asano, M.2022 | Low | Some concerns | Low | Low | Low | Some concerns | 1 |
| 6 | Nahra, R.2021 | Low | Low | Low | Low | Low | Low | 1 |
| 7 | Saxena, A. R.2023 | Low | Low | Low | Low | Low | Low | 1 |
| 8 | Saxena, A. R.2023 | Low | Some concerns | Low | Low | Low | Some concerns | 1 |
| 9 | Camastra, S.2017 | Low | Some concerns | Low | Low | Low | Some concerns | 1 |
| 10 | Dutour, A.2016 | Low | Low | Low | Low | Low | Low | 1 |
| 11 | Siskind, D. J.2018 | Low | Some concerns | Low | Low | Low | Some concerns | 1 |
| 12 | Alba, M.2021 | Low | Low | Low | Low | Low | Low | 1 |
| 13 | Di Prospero, N. A.2021 | Low | Some concerns | Low | Low | Low | Some concerns | 1 |
| 14 | Astrup, A.2012 | Low | Low | Low | Low | Low | Low | 1 |
| 15 | Astrup, A.2009 | Low | Low | Low | Low | Low | Low | 1 |
| 16 | Blackman, A.2016 | Low | Some concerns | Low | Low | Low | Some concerns | 1 |
| 17 | Davies, M. J.2015 | Low | Low | Low | Low | Low | Low | 1 |
| 18 | Dejgaard, T. F.2016 | Low | Low | Low | Low | Low | Low | 1 |
| 19 | Dejgaard, T. F.2020 | Low | Some concerns | Low | Low | Low | Some concerns | 1 |
| 20 | Ghanim, H.2020 | Low | Some concerns | Low | Low | Low | Some concerns | 1 |
| 21 | Halawi, H.2017 | Low | Low | Low | Low | Low | Low | 1 |
| 22 | Kim, S. H.2013 | Low | Some concerns | Low | Low | Low | Some concerns | 1 |
| 23 | Larsen, J. R.2017 | Low | Low | Low | Low | Low | Low | 1 |
| 24 | Lind, M.2015 | Low | Some concerns | Low | Low | Low | Some concerns | 1 |
| 25 | O'Neil, P. M.2018 | Low | Low | Low | Low | Low | Low | 1 |
| 26 | Pi-Sunyer, X.2015 | Low | Some concerns | Low | Low | Low | Some concerns | 1 |
| 27 | Robert, S. A.2015 | Low | Some concerns | Low | Low | Low | Some concerns | 1 |
| 28 | Schmidt, S.2022 | Low | Low | Low | Low | Low | Low | 1 |
| 29 | Smits, M. M.2016 | Low | Some concerns | Low | Low | Low | Some concerns | 1 |
| 30 | van Eyk, H. J.2020 | Low | Some concerns | Low | Low | Low | Some concerns | 1 |
| 31 | Whicher, C. A.2021 | Low | Low | Low | Low | Low | Low | 1 |
| 32 | Jastreboff, A. M.2023 | Low | Some concerns | Low | Low | Low | Some concerns | 1 |
| 33 | Rosenstock, J.2023 | Low | Some concerns | Low | Low | Low | Some concerns | 1 |
| 34 | Frias, J. P.2023 | Low | Low | Low | Low | Low | Low | 1 |
| 35 | Wharton, S.2023 | Low | Some concerns | Low | Low | Low | Some concerns | 1 |
| 36 | Blundell, J.2017 | Low | Low | Low | Low | Low | Low | 1 |
| 37 | Davies, M.2021 | Low | Low | Low | Low | Low | Low | 1 |
| 38 | Mu, Y.2023 | Low | Low | Low | Low | Low | Low | 1 |
| 39 | Wilding, J. P. H.2021 | Low | Low | Low | Low | Low | Low | 1 |
| 40 | Marso, S. P.2016 | Low | Low | Low | Low | Low | Low | 1 |
| 41 | Garvey, W. T.2022 | Low | Low | Low | Low | Low | Low | 1 |
| 42 | Kadowaki, T.2022 | Low | Low | Low | Low | Low | Low | 1 |
| 43 | Sorli, C.2017 | Low | Some concerns | Low | Low | Low | Some concerns | 1 |
| 44 | Zinman, B.2019 | Low | Some concerns | Low | Low | Low | Some concerns | 1 |
| 45 | Wharton, S.2023 | Low | Low | Low | Low | Low | Low | 1 |
| 46 | Lingvay, I.2018 | Low | Low | Low | Low | Low | Low | 1 |
| 47 | Davies, M.2017 | Low | Low | Low | Low | Low | Low | 1 |
| 48 | Pratley, R.2019 | Low | Low | Low | Low | Low | Low | 1 |
| 49 | Dahl, D.2022 | Low | Low | Low | Low | Low | Low | 1 |
| 50 | Garvey, W. T.2023 | Low | Low | Low | Low | Low | Low | 1 |
| 51 | Jastreboff, A. M.2022 | Low | Low | Low | Low | Low | Low | 1 |
| 52 | Rosenstock, J.2021 | Low | Low | Low | Low | Low | Low | 1 |
| 53 | Ji, L.2021 | Low | Low | Low | Low | Low | Low | 1 |
| 54 | Ji, L.2023 | Low | Low | Low | Low | Low | Low | 1 |
| 55 | Urva, S.2022 | Low | Low | Low | Low | Low | Low | 1 |

# Figure S2. The summary risk of literature assessment


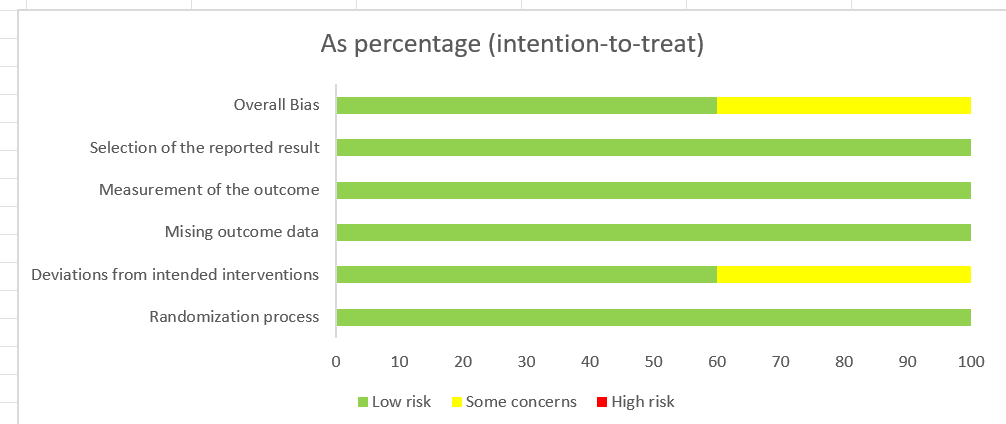


# Table S6. Parameter estimates of the final model

|  | **Final model** | | **SIR** | |
| --- | --- | --- | --- | --- |
|  | Value | RSE(%) | Median | 95%CI |
| Structure parameter |  |  |  |  |
| E_max_BI 456906_,kg | -13.5 | 14.8 | -13.8 | -17.9, -9.66 |
| E_max_Cotadutide_,kg | -10.5 | 14.8 | -10.6 | -13.3, -7.86 |
| E_max_Danuglipron_,kg | -9.29 | 24.7 | -9.37 | -13.9, -5.30 |
| E_max_Exenatide_,kg | -6.05 | 8.60 | -6.10 | -7.09, -5.12 |
| E_max_JNJ-64565111_,kg | -18.6 | 6.10 | -18.7 | -20.7, -16.3 |
| E_max_Liraglutide_,kg | -4.25 | 9.30 | -4.26 | -5.08, -3.46 |
| E_max_Retatrutide_,kg | -22.6 | 19.3 | -22.6 | -30.9, -15.2 |
| E_max_Orforglipron_,kg | -14.7 | 7.40 | -14.6 | -16.7, -12.4 |
| E_max_Semaglutide(INJ)_,kg | -11.7 | 12.0 | -11.7 | -14.3, -8.81 |
| E_max_Semaglutide(P.O)_,kg | -5.36 | 22.8 | -5.41 | -7.88, -3.16 |
| E_max_Tirzepatide_,kg | -12.9 | 9.10 | -12.9 | -15.4, -10.6 |
| E_max_Mazdutide_,kg | -7.75 | 17.9 | -7.73 | -10.8, -5.11 |
| k,1/week | 0.0578 | 8.60 | 0.0572 | 0.0480, 0.0666 |
| θAge on E_max_ | -0.0304 | 23.0 | -0.0306 | -0.0428, -0.0160 |
| θDose on E_max_Cotadutide_ | 0.219 | Fixed | - | - |
| θDose on E_max_Danuglipron_ | 80 | Fixed | - | - |
| θDose on E_max_JNJ-64565111_ | 6.73 | Fixed | - | - |
| θDose on E_max_Retatrutide_ | 4 | Fixed | - | - |
| θDose on E_max_Orforglipron_ | 14.6 | Fixed | - | - |
| θDose on E_max_Semaglutide(INJ)_ | 0.384 | Fixed | - | - |
| Inter-study variability | | | | |
| ηE_max_ | 0.330 | 11.1 | 0.339 | 0.271, 0.404 |
| ηk | 0.627 | 11.1 | 0.626 | 0.479, 0.744 |
| Residual error |  | | | |
| ε | 0.391 | 6.30 | 0.389 | 0.342, 0.440 |

E_max_ denotes the maximal effect of the drug; k represents the rate of drug onset, and 0.693/k indicates the time required to achieve 50% of E_max_. ‘θAge on E_max_’refers to the covariate parameter assessing the impact of age on E_max_. ‘θDose on E_max_’ indicates the effect of dose on E_max_. η represents the inter-study variability of the model parameter, and ε is the residual error. CI stands for confidence interval, and RSE is the relative standard error.

# Figure S3. Onset times of different GLP-1 receptor agonists


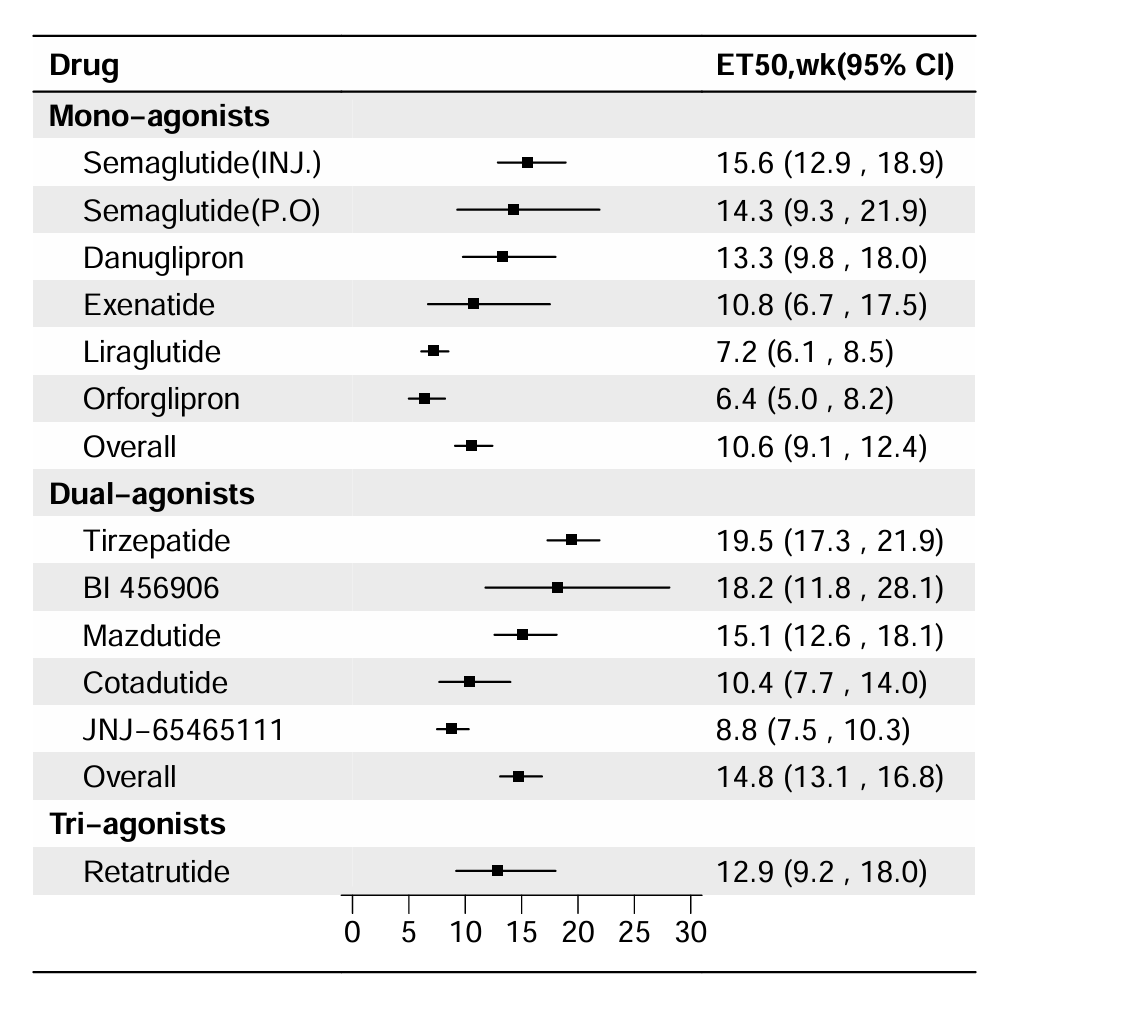


ET_50_ represents the time required to achieve 50% of the maximal effect, calculated as ET_50_ = 0.693/k, where k denotes the rate of onset. The k values for each drug and their 95% CIs were obtained using Bayesian feedback combined with single-arm meta-analysis

# Figure S4. The goodness-of-fit plots of the final model


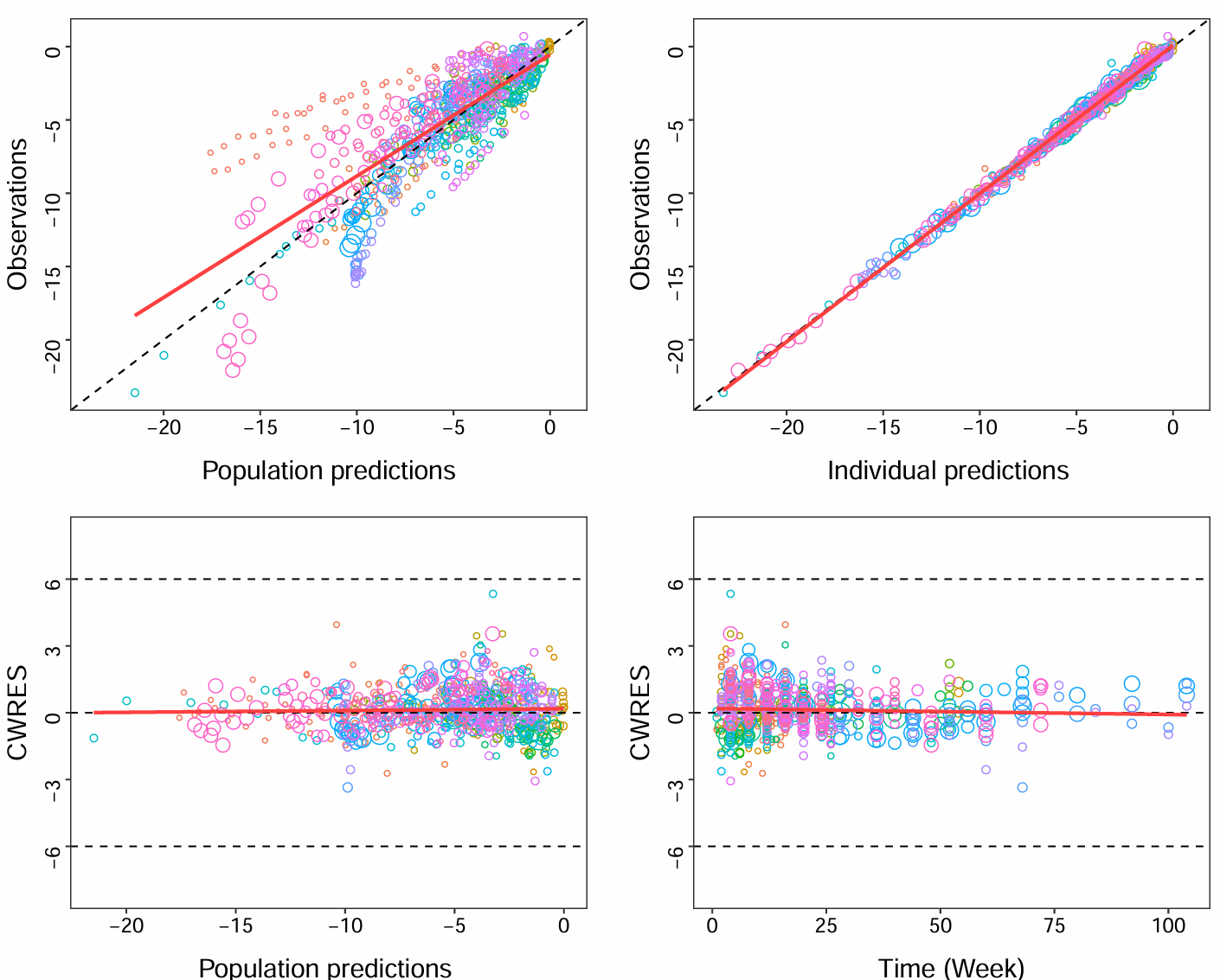


The upper left panel of the figure illustrates the relationship between population predicted values and observed data, with a black line indicating the line of unity and a solid line representing the line of best fit. The upper right panel depicts the correlation between individual predicted values and observed data, also with a black unity line and a red line of best fit. In the lower left panel, conditional weighted residual errors (CWRES) are plotted against population prediction values, where the black line represents the zero residual error line. The lower right panel presents CWRES versus time, with similar dashed and solid line representations.

# Figure S5. Correlation plot of model parameters and covariates


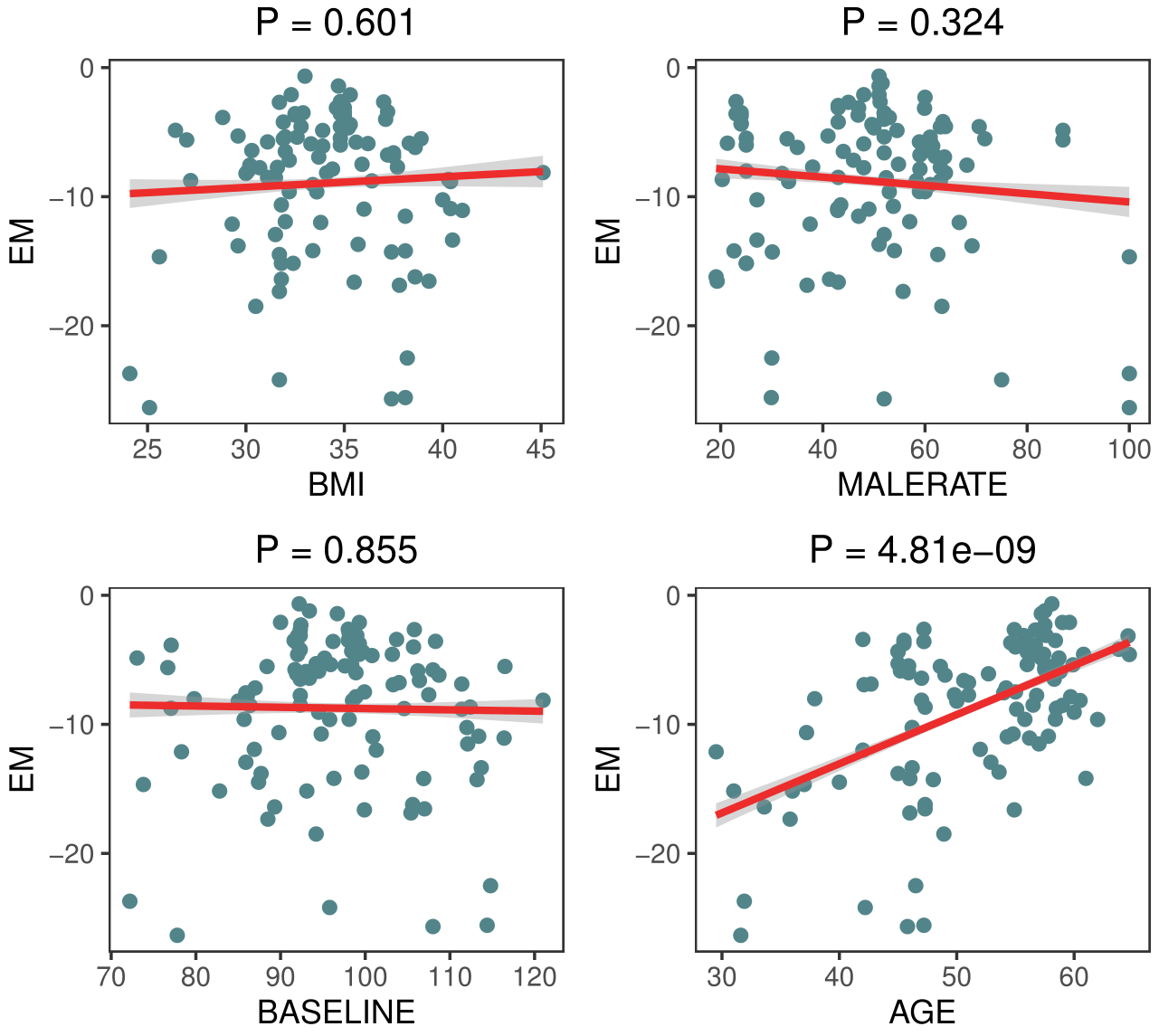


Pearson correlation analysis was employed to assess the relationship between continuous variables and the model parameter E_max_. An association was considered statistically significant if the P-value was less than 0.05. EM represents the maximum effect of the drug (E_max_).

# Methods 1. Model building

Base model establishment

Exploratory data analysis revealed that the ΔΔWeight in the drug group increased over time, eventually reaching a plateau of drug efficacy. This characteristic of the data can be described using the following model.

$\boldsymbol{E}_{\boldsymbol{i,j}}\boldsymbol{=}\boldsymbol{E}_{\boldsymbol{max,i}}\boldsymbol{\times}\left( \boldsymbol{1-}\boldsymbol{e}^{\boldsymbol{-}\boldsymbol{k}_{\boldsymbol{i}}\boldsymbol{\times}\boldsymbol{time}_{\boldsymbol{j}}} \right)\boldsymbol{+}\frac{\boldsymbol{\varepsilon}_{\boldsymbol{i,j}}}{\sqrt{\boldsymbol{N}_{\boldsymbol{i,j}}}}$ Equation 1

$\boldsymbol{E}_{\boldsymbol{max,i}}\boldsymbol{=}\boldsymbol{E}_{\mathbf{max}\boldsymbol{\_typical}}\boldsymbol{\times}\boldsymbol{e}^{\boldsymbol{\eta}_{\boldsymbol{Emax}}}$ Equation 2

$\boldsymbol{k}_{\boldsymbol{i}}\boldsymbol{=}\boldsymbol{k}_{\boldsymbol{typical}}\boldsymbol{\times}\boldsymbol{e}^{\boldsymbol{\eta}_{\boldsymbol{k}}}$ Equation 3

In Equation 1, E_i,j_ represents the efficacy value for trial group *i* at observation time point *j*, E_max,i_ is the maximum efficacy for trial group *i*, and k_i_ is the onset rate for trial group *i*, with the time required to reach 50% of maximum efficacy being 0.693/k_i_.

Time_j_ is the time at the *j^th^* observation time point, measured in weeks. ε_i,j_ represents the residual variability for trial group *i* at observation time point j, which follows a normal distribution with a mean of 0 and a variance of 𝜎^2^, and is weighted by the inverse of the square root of the sample size, meaning that a larger sample size results in smaller residual variability. In Equations 2-3, 𝐸_max,typical_ is the typical value for the parameter 𝐸_max_, and 𝑘_typical_ is the typical value for the parameter 𝑘. 𝜂_𝐸max_ and 𝜂_𝑘_ are the inter-trial variabilities for 𝐸_max_ and 𝑘, respectively, and both follow normal distributions centered at 0 with variances of 𝜔_1_^2^ and 𝜔_2_^2^, respectively.

For drugs exhibiting a dose-response effect, the relationship between dose and effect will be described using the following model.

$\boldsymbol{E}_{\boldsymbol{max,}\boldsymbol{dose}\boldsymbol{,i}}\boldsymbol{=}\boldsymbol{E}_{\boldsymbol{max}}\boldsymbol{\times}\frac{\boldsymbol{Dose}_{\boldsymbol{i}}}{\boldsymbol{ED}_{\boldsymbol{50}}\boldsymbol{+}\boldsymbol{Dose}_{\boldsymbol{i}}}$ Equation 4

In Equation 4, 𝐸_max,dose_ represents the 𝐸max value of a drug at a given Dose_i_ , and

ED_50_ is the dose required for the drug to achieve 50% of its E_max_ value. Where data conditions permit, this study will estimate the E_max_ values, *k* values, and ED_50_ values for each drug individually. If the conditions are not met, parameters will be estimated using shared values. Subsequently, the parameters for each drug will be calculated using Bayesian feedback combined with a single-arm meta-analysis.

Covariate model establishment

After establishing the base model, potential covariates that may influence model parameters can be examined, including participant age, male ratio, baseline weight, and baseline BMI, among others. The method for incorporating variables is shown in Equations 5-7.

$\boldsymbol{P}_{\boldsymbol{cov}}\boldsymbol{=}\boldsymbol{P}_{\boldsymbol{typical}}\boldsymbol{+(COV-}\boldsymbol{COV}_{\boldsymbol{median}}\boldsymbol{)\times}\boldsymbol{\theta}_{\boldsymbol{COV}}$ Equation 5

$\boldsymbol{P}_{\boldsymbol{cov}}\mathbf{=}\boldsymbol{P}_{\boldsymbol{Typical}}\boldsymbol{\times}\left( \boldsymbol{COV}\mathbf{/}\boldsymbol{COV}_{\boldsymbol{median}} \right)^{\boldsymbol{\theta}_{\boldsymbol{COV}}}$ Equation 6

$\boldsymbol{P}_{\boldsymbol{cov}}\mathbf{=}\boldsymbol{P}_{\boldsymbol{Typical}}\boldsymbol{\times}\boldsymbol{e}^{\boldsymbol{(COV-}\boldsymbol{COV}_{\boldsymbol{median}}\boldsymbol{)\times}\boldsymbol{\theta}_{\boldsymbol{COV}}}$ Equation 7

In Equations 4 to 7, 𝑃_cov_ represents the parameter value corresponding to a specific level of a covariate, COV is the value of the covariate, and COV_median_ is the median value of the covariate in the population. P_typical_ is the typical value of the parameter when the covariate equals COV_median_. θ_COV_ is the correction coefficient for the covariate effect on the model parameter.

This study introduced covariates into the base model using a forward-backward procedure for analysis. Whether a covariate is retained in the final model depends on the results of likelihood ratio tests and its ability to effectively reduce inter-trial variability of model parameters. During the model inclusion process, the statistical significance level was set at 0.05, with a corresponding threshold decrease in the objective function value (OFV) of 3.84 (the critical value of a chi-squared distribution with one degree of freedom for P<0.05). In the model exclusion process, the significance level was adjusted to 0.01, and the threshold increase in OFV was set at 6.63 (the critical value of a chi-squared distribution with one degree of freedom for P<0.01). Additionally, the interpretability of the included covariates was considered to determine if they should be retained in the final model.

# Methods 2. Model evaluation

This study evaluated the quality of the model using the following methods:

1) Diagnostic Plot Analysis: The predictive accuracy of the model was assessed by comparing population predictions, individual predictions, and observed values. Weighted residual scatter plots against time and population observations were created to check the random distribution of conditional weighted residuals, thereby verifying the reasonableness of the model assumptions.

2) Sampling importance resampling (SIR) method: It was used for repeated sampling (1000 times) to obtain the median value of the distribution of the model parameters and their 95% confidence interval, which will then be compared with the model parameters obtained from the original dataset.

3) Prediction-corrected Visual Predictive Check (pc-VPC): Based on the final model parameters, 1000 Monte Carlo simulations were performed to generate simulated efficacy datasets. The prediction intervals were standardized to enhance the ability to identify model discrepancies. By comparing the statistical quantiles between the simulated data and the original data, the central tendency and variability were analyzed, thus evaluating whether the model accurately reflects the characteristics of the data distribution and further validating the model's predictive performance.
